# Non-destructive fluorescence sensing for assessing microclimate, site and defoliation effects on flavonol dynamics and sugar prediction in Pinot blanc grapes

**DOI:** 10.1371/journal.pone.0273166

**Published:** 2022-08-16

**Authors:** Selena Tomada, Giovanni Agati, Enrico Serni, Samanta Michelini, Valentina Lazazzara, Ulrich Pedri, Christof Sanoll, Aldo Matteazzi, Peter Robatscher, Florian Haas

**Affiliations:** 1 Laimburg Research Centre, Laimburg, Italy; 2 Faculty of Science and Technology, Free University of Bozen-Bolzano, Bolzano, Italy; 3 Istituto di Fisica Applicata ‘Nello Carrara’, Consiglio Nazionale delle Ricerche (CNR), Sesto Fiorentino, Italy; Universidade do Minho, PORTUGAL

## Abstract

In an era of growing international competition in modern viticulture, the study and implementation of innovative technologies to increase the production of high-quality grapes and wines are of critical importance. In this study, the non-destructive portable sensor Multiplex, based on fluorescence sensing technique, was applied to evaluate grape maturity parameters and flavonol content of the understudied Pinot blanc variety. The effects of environmental and agronomical factors on flavonol content of Pinot blanc grapes were investigated in eight vineyards characterised by different microclimatic and agronomic conditions. Furthermore, the direct impact of canopy management treatment on the flavonol dynamics of the grapes oriented in the four cardinal directions was assessed. Results highlight the positive role of moderate temperatures and direct sunlight exposure on Pinot blanc flavonol content; however, no direct vineyard-elevation effect was observed. The ability to modulate and evaluate the flavonol content in field represent crucial factors because of their potential effect on flavonoids-dependent wine characteristics, such as stability and ageing. In the present study, for the first time, two calibration curves were reported for pre- and post-veraison periods between flavonol indices and the berry skin flavonol content and a good correlation was observed between Multiplex measurement and the total polyphenolic content of grape juice. Moreover, the strong correlation between the chlorophyll index with grape juice sugar content and titratable acidity revealed the practical application of non-destructive sensors to predict the optimal harvest time for Pinot blanc grapes. In conclusion, the non-destructive fluorescence sensor Multiplex is a high-potential tool for innovative viticulture, for evaluating grape skin composition variables in white grape varieties.

## Introduction

Pinot blanc is an important white grape variety used for the production of dry neutral wines or as a valuable ingredient in sparkling wines. In Europe, it is grown mainly in Germany, Italy, Austria, and France [1,2]. The total surface of Pinot blanc vineyards has now risen to 15 thousand hectares worldwide [2]. Although Pinot blanc is getting attention in the global wine market, the characterisation of this variety is still at the early stages. It has been recognized to be an independent somatic mutation of Pinot noir due to deletion of *VvMybA1* and *VvMybA2* genes [3], which are reported to be involved in the regulation of anthocyanin biosynthesis in *V*. *vinifera* grapes [4]. The studies on Pinot blanc focus mainly on the classification of Pinot grape-berry skin-colour mutant via microsatellite analysis [3,5] and on the nutraceutical properties of the grape by-products derived from the winery industry [6,7]. Therefore, only little information about the Pinot blanc grape composition, and specifically on the flavonoids profile, is available so far [8,9]. The grapevine flavonoids are secondary metabolites present mainly in the skin and seeds of grape berries, synthesised along the general phenylpropanoid pathway [10,11]. Among them, the most relevant compounds of grape varieties are represented by anthocyanins, flavanols, flavonols, and proanthocyanidins [12,13]. In the winemaking sector, flavonoids are known to be important compounds contributing to perceived wine quality and style [14]. They are responsible for organoleptic characteristics such as colour, astringency, and wine bitterness; for instance, flavonols are involved in the colour-enhancement of red wine due to co-pigmentation, while their antioxidant activity is considered particularly important for wine-ageing, especially in white-wines [15–17]. Nevertheless, flavonols provide a variety of positive effects on human health (*e*.*g*., antimicrobial, anti-aging, anti-inflammatory) [18,19].

Flavonol glycosides, which occur in the outer layers of both red and white grape skin, act as UV-screening molecules and free radical scavengers. Their biosynthesis, even if under genetic control, can be affected by the grape berries’ microclimate conditions [20–22]. Indeed, berry flavonols were found to increase their concentration proportionally to the bunch sunlight exposure [23] so that they can be considered as bio-actinometers.

The standard wet chemistry methods usually applied to investigate the polyphenolic composition of the grape are destructive, costly, time consuming, and they are based on a limited number of samplings that may not be fully representative of the whole grape production in a specific time-point. Therefore, modern viticulture is evolving by the evaluation of several agronomic *in-planta* parameters directly in the vineyards, as decision support tools for winemakers. In this way, portable optical sensors based on non-destructive fluorescence measurements have been developed in order to estimate grape maturity through the evaluation of some classes of flavonoids accumulated in the berry skin during ripening [24,25]. This technique was developed mainly to determine the phenolic maturity of red grapes by the evaluation of their anthocyanin content [26], and only few cases regarding the application of this method on white grapes are reported [25,27,28]. Specifically, the method relies on the measurement of chlorophyll fluorescence under different excitation wavelengths, including those corresponding to the absorbance of flavonol glycosides, in order to provide a flavonol prediction index. Besides flavonols, the fluorescence-based sensors provide indices of berry chlorophyll content which can precisely mark veraison and can be used as maturity indicator in white grape by inverse correlation to the berry sugar content [25,27]. These optical sensors are therefore useful to properly follow the grape ripening process in the field by providing real-time data, by measuring a large number of samples representative of whole plots, and by measuring kinetics on the very same bunches that would reduce effects due to the inter-vines and intra-plant variability.

The aim of this study was to apply the above non-destructive technique for evaluating the effect of winegrowing practises, microclimatic vineyard conditions, and sites on the flavonol and sugar content of Pinot blanc grape cultivated in different areas of South Tyrol (Italy) during the berry development period. In particular, the technique allowed the detection of temporal dynamics of flavonols and Total Soluble Solids (TSS) concentration on grapes in vineyards at different locations and elevations, during two consecutive seasons, and under defoliation treatments. The correlation between the non-destructive optical indices and the analytical data from destructive analyses of grape berries was also investigated.

## Materials and methods

### Vineyards under study

The field trials were carried out in South Tyrol, northern Italy. Eight private vineyards, homogeneously distributed in the Adige Valley and located in four winegrowing zones (municipalities): Eppan (Ep), Nals (Na), Terlan (Te), and Tramin (Tr), were chosen. The vineyards were comparable for variety (Pinot blanc cv.), rootstock (SO_4_), plant age (10 years), training system (espalier with Guyot pruning), and vine spacing (0.7 m x 2.0 m). Two vineyards for each place, one located in the valley (-1) and the other along the hill (-2), were selected. The vineyards elevation ranged from 223 to 730 m a.s.l. Each vineyard was named with an abbreviation containing the municipality’s name and the location’s number. In the vineyards: Na-1, Te-1, Te-2, and Tr-2, the grapevine rows were east-west oriented, so that grape bunches were located on the north (N) and south (S) sides of the rows. In the vineyards: Ep-1, Ep-2, Na-2, and Tr-1, the grapevine rows were north-south oriented, therefore, the bunches were located on the east (E) and west (W) sides of the rows. The study was conducted in 2017 and 2018. The PinotBlanc project was developed by the Laimburg Research Centre in collaboration with the South Tyrolean Wine Consortium, with direct participation from Tramin, Appiano, Terlano, Nalles-Margreid, and the Gummererhof winery, which provided access to the private vineyards under investigation.

### Climatic and agronomic data

Temperature data were recorded for each vineyard over the vegetative seasons for the period under study. Air temperature sensors were allocated 2 m above ground and provided hourly recorded data used to calculate the average, minimum, and maximum daily temperature. Missing values were replaced with predicted values provided by a forecasting model specifically generated for the South Tyrol region [29]. Before processing, data were analysed and compared for reliability with the available sensor data. Two bioclimatic indices were derived from the temperature data: i) Growing Degree Days (GDD) [30]; ii) Grapevine Sugar Ripeness (GSR) [31], and they were calculated over veraison-technological maturity period, using a base temperature of 10°C and 0°C, respectively. The grapevine phenological stages were dated according to the international BBCH scale [32], following the standard guidelines of the Organisation Internationale de la Vign et du Vin [33]: budburst (BBCH 7); flowering (BBCH 65); bunch-closure (BBCH 77); veraison (BBCH 81); ripening (BBCH 89).

The vineyard vigour was evaluated as shoot growth rate (SGR) and, specifically, the number of new growing leaves was assessed weekly from bunch closure to veraison on five shoots *per* plant, with three replicates, each made of three plants *per* vineyard. The mineral soil composition of each vineyard was evaluated, and the sampling was made by soil corer at a depth from 10 to 60 cm. Multiple and randomised samples were taken to obtain 500 g of soil *per* replicate, and sampling was performed in triplicate. The pH, macro and micro element were analysed following the standard methods: DIN ISO 10694:1995; ÖNORM L 1087:2012; DIN EN 15933:2012. The monthly potential solar radiation for each site was provided by the model of Egarter Vigl *et al*. [29]. Data about the transpiration efficiency, used as an indicator of the drought stress, were evaluated *via* the stable carbon isotopic fraction ^13^C/^12^C of the ethanol of wines produced *via* microvinification of grapes harvested from the vineyards under investigation [34], following the standard method OIV-MA-AS312-06 R2001 (EA-IRMS) (Reg. CE 2676/1990; Reg. CE 440/2003). The isotopic analyses were carried out by the external services of the Laboratorio dell’Unità Chimica Vitienologica e Agroalimentare, Fondazione Edmund Mach—ACCREDIA 0193 (TN, Italy) and data provided by Michelini *et al*. [34].

### Spectroscopic indices

The spectroscopic indices of flavonol compounds (FLAV and FLAV_UV) and chlorophyll (SFR_R) were assessed on the skin of intact berries using the Multiplex® 3.6 (Mx) commercial optical device (Force-A, Orsay, France) as Mx units. The indices are defined by the following formulas: FLAV = log(FRF_R/FRF_UV) [35], FLAV_UV = log(1/FRF_UV) [27] and SFR_R = FRF_R/RF_R [26]. Specifically, FRF_UV and FRF_R are the chlorophyll fluorescence emitted in the far-red region (far-red fluorescence, FRF) under the excitation with UV at 375 nm and red (R) at 630 nm radiation, respectively. The RF_R is the chlorophyll red fluorescence excited by R. Before calculations, the signals were corrected for the electronic noise and normalised to a fluorescence standard (blue plastic foil) following the manufacturer’s instruction. The sensor indices were evaluated in every experiment unless otherwise specified.

### *In situ* monitoring of berry ripening and flavonol content

The phenolic maturity was assessed by the Mx optical device in sunny and dry weather condition, approximately every 15 days from grapes veraison until the period close to technological maturity, in two consecutive vintages, specifically DOY 201, 212, 228, 242, 256 in 2017 and DOY 200, 214, 228, 243, 254 in 2018. In 2018, a pre-veraison measurement, corresponding to the bunch-closure stage, was added (DOY 185). Specifically, the samplings can be grouped in six time-points: 0, DOY 185; 1, DOY 200–201; 2, DOY 212–214; 3, DOY 228; 4, DOY 242–243; 5, DOY 254–256. In each of the eight vineyards under investigation, three rows, one located at the top, one in the middle, and one at the bottom of a 1000 m^2^ field-plot were selected, and 50 bunches/row (technical replicates) were optically measured *per* day of sampling. The 50 measurements were equally distributed between the two row sides. A farmer-standard defoliation consisting in a partial defoliation of the grape-bunches area (30% of the leaves were retained) was applied seven days after the full-bloom (BBCH 65). The technological maturity parameters were destructively assessed on the same days of the Mx measurements. Specifically, three replicates per vineyard made by 50 berries each were randomly sampled, at the bottom, central, and top sides of the bunches, and crushed into juice that was subsequently analysed for total soluble solids (TSS expressed in °Babo) and acidity parameters [titratable acidity (g/L), malic acid, and tartaric acid (g/L)] by a spectroscopic method (FT-IR WineScan, FOSS, Denmark).

### Defoliation trial

To investigate the effects of agronomical practises on the berry-skin flavonol accumulation in Pinot blanc, Ep-1 and Te-1 vineyards, respectively N-S and E-W oriented, were selected. To evaluate the natural leaf shading effect, an intense defoliation (ID) treatment, consisting in a total leaf-removal treatment on both sides of the row in the grapes zone (30 cm diameter around the bunch), was applied one week after the full-bloom (DOY 163), and compared with the non-defoliated (ND) control treatment. The optical sampling was performed on the E and W row-sides in Ep-1 and on the N and S row-sides in Te-1. An example of the corresponding acronym of the treatment is made as follow: field name, grape row-localisation, treatment (e.g., Ep-W-ID). Five Mx temporal measurements were carried out from bunch-closure to grape technological maturity in 2018 (DOY: 185, 200, 214, 228, 243). Each optical sampling was composed of three biological replicates, each one made of 25 flashed bunches (technical replicates). Data of global solar radiation (W/m^2^) of the bunches-zone, in ID and ND treatments, were recorded hourly on both sides of the row by a BPW34 photodiode sensor (Vishay Intertechnology, Lancaster, PA, USA). The inside bunch-temperature was recorded hourly by the DS18b20 micro-temperature sensor (Maxim Integrated, San Jose, CA, USA). The global solar radiation sensors were calibrated with data retrieved from the provincial weather station no. 85700MS (46.3825° - 11.2887°, Laimburg, BZ, www.meteo.provincia.bz.it). At DOY 243 according to the treatment, three replicates were randomly sampled and crushed into juice. The juice was filtrated (0.45 μm) and the total polyphenolic content (mg/L) was determined by the Folin–Ciocalteu method [36] using a Hyperlab wine analyzer (Steroglass, Italy).

### Destructive analyses

#### Sampling and flavonol extraction

Berries samples were collected in the 2018 vintage from DOY 186 to DOY 246 from a single vineyard (Te-1, 270 m a.s.l.). The veraison stage was recorded at DOY 202. In total, 33 samples divided into seven sampling dates were analysed, three performed from DOY 186 to DOY 208 in the bunch closure-veraison period, and the others during the berry ripening from DOY 212 to DOY 246. The berries corresponding to the area exposed to the Mx readings were sampled separately. Each sample was composed of 20–25 berries that were subsequently stored at -30°C prior to analysis. Flavonol glycosides were extracted from grape skin [37]. Briefly, grape skins were separated from pulp and dried at 45°C, ground to a fine powder using a mixer-mill disruptor (MM301 Retsch) for 150 sec at 30 Hz, and weighed (20.0 mg) in plastic tubes. The powder was spiked with internal standard (quercetin-4-glucoside), then extracted with 12.0 mL of methanol-water mixture (1:1) using a rotary mixer and ultrasonic bath in sequence (total time: 30 min). Finally, samples were centrifuged, and supernatants were transferred in plastic tubes and stored (-30°C) until analysis.

#### UltraViolet-Visible Spectroscopy analysis

The UltraViolet-Visible Spectroscopy (UV/Vis) analysis of berry skin extracts was conducted on a Cary 60 UV/Vis spectrophotometer (Agilent, Santa Clara, US). Quartz cuvettes (1.0 cm path length) were used. Absorbance of extracts was measured at 375 nm (slit 2 nm) and spectra were recorded in the 200–700 nm range. Absorbance spectra of standard compounds were also recorded in the 200–400 nm range.

#### Liquid chromatography–mass spectrometry analysis

High-performance liquid chromatography-mass spectrometry (HPLC-MS) analysis was performed on skin extracts [37]. Briefly, phenolics were separated on a RSLC Acclaim C18 column (100 × 2.1 mm, 2.2 μm particle size, Thermo Fisher Scientific, Waltham, US) with mobile phase consisting of 0.1% formic acid in water (A), and 0.1% formic acid in acetonitrile (B), with 0.5 mL/min flow rate, 40°C column temperature and 5 μL injection volume. Quali-quantitative determination of analytes was achieved with TSQ Quantum Access Max triple-quadrupole mass spectrometer equipped with heated electrospray ionisation (HESI) source (Thermo Fisher Scientific, Waltham, USA). Mass spectrometry data were acquired in positive mode for all analytes. Collision-induced fragmentation was obtained with argon (1.5 m Torr pressure) and collision energy optimised for each compound. Selected reaction monitoring (SRM) was applied using two mass fragments for each compound and discrimination of isomers (same parent and product ions) was obtained by their retention times (Rt). On-line UV-Vis detection was also used (360 nm wavelength), and UV-VIS spectra in the range 200–750 nm were acquired during the whole run. Quantitative determination was based on comparison of analyte/internal standard ratio in samples and calibration solutions (average of triplicates), with external calibration curve for each reference compound.

### Statistical analysis

Correlations between: (i) spectroscopic indices (FLAV, FLAV_UV and SRF_R) and berry skin flavonol content and TSS analytically measured; (ii) total flavonol glycosides (LC-MS) and absorbance (at 375 nm); (iii) FLAV and environmental factors (cumulative irradiance; average (avg) inside-bunch temperature) or grape juice composition (total polyphenolic content) were evaluated by linear regressions and the R^2^ coefficient.

The data of the two vintages under investigation were separately analysed, unless otherwise specified. In pairwise comparison, Student’s *t*-test was applied to compare FLAV at each time-point between vintages and to discriminate the defoliation trail parameters within grape row-localisation. To investigate differences between vineyards, the data of grape maturity parameters and Mx indices for the defoliation trial were firstly checked for normality and homoscedasticity and then subjected to one-way ANOVA, where mean comparisons were performed with Tukey’s test (α = 0.05). If the assumptions were not matched, mainly due to the different number of observations (effect of grape-bunch localisation and site-elevation on FLAV), the data were subjected to Kruskal-Wallis test, where mean comparisons were performed with Dunn Bonferroni test (α = 0.05). The statistical tests were carried out using SPSS Statistics 24 (IBM, Italy).

Correlations between flavonol content and agro-climatic variables and the overall variation in the data were examined by Principal Component Analysis (PCA). PCA of agro-climatic parameters and flavonol content (FLAV, time-point 4 averaged on three replicates) at different grape bunch expositions, i.e., exposed (south-east) and covered (north-west), was computed on data collected during the two vintages for each site, except for Te-2 2018. Agro-climatic variables consist of SGR, soil mineral compounds [i.e., nitrogen (Soil_N), phosphorus (Soil_P) and potassium (Soil_KCO)], cumulative temperatures from veraison to harvest [average (Tavg), minimum (Tmin), maximum (Tmax), temperature range (Trange) and day with temperature above 35°C (DD35)], GDD, potential solar radiation, stable carbon isotopic fraction (^13^C/^12^C), field slope, and site elevation. The R package *FactoMineR* [38] was used for the multivariate analyses on scaled variables, and the *factorextra* package [39] was used for the visualisations of the loading vectors and the principal component scores plot, which project the original variables and observations, respectively, onto a pair of PCs. The biplot combines both the principal component scores and the loading vectors by representing observations in the new 2-dimensional space defined by those PCs.

## Results and discussion

### Calibration curves for the optical indices

**Fig 1:**
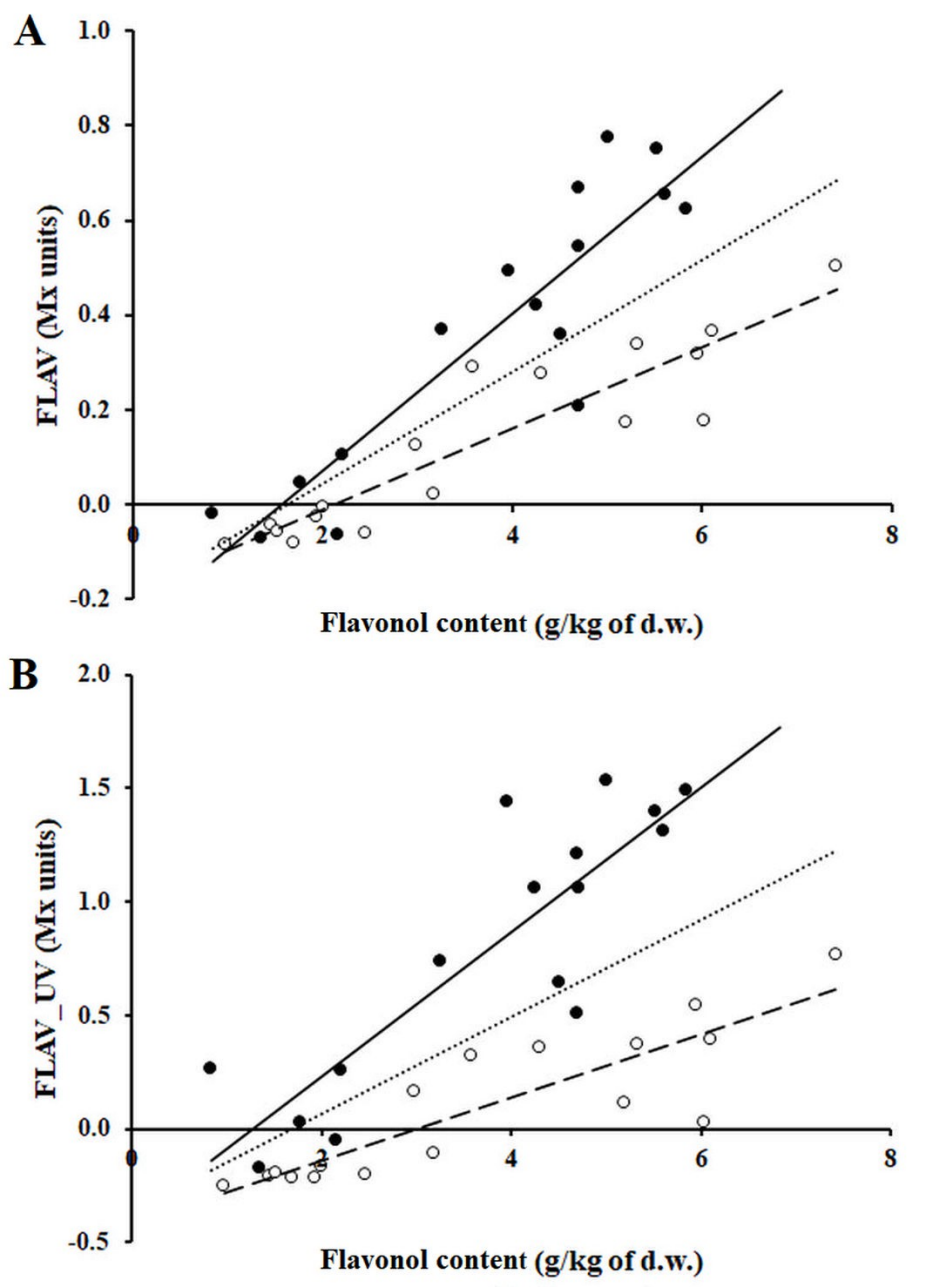
Correlation curve between spectroscopic indices and the berry skin flavonol concentration measured analytically. The Multiplex measurements were taken in 2018 at the Tramin valley vineyard (north-south oriented). The total flavonol compounds were extracted from the skin of previously Mx flashed berries and analysed by HPLC-MS. The wet chemistry data were correlated to FLAV (A) and FLAV-UV (B) indices. Empty (○) and solid (●) dots represent pre- and post-veraison samples, respectively. Trend lines for the whole set of data (dotted-line) are displayed, the fitting curve equations are: A) FLAV, y = 0.1181x – 0.1923, R^2^ = 0.62 and B) FLAV_UV, y = 0.2126x – 0.3554, R^2^ = 0.43. The equations for groups of data are: A) y = 0.0859x – 0.1818, R^2^ = 0.84 and y = 0.1654x – 0.2572, R^2^ = 0.81; B) y = 0.1383x – 0.4143, R^2^ = 0.76 and y = 0.3175x – 0.4004, R^2^ = 0.75, for pre- (dashed line) and the post-veraison (solid line) periods, respectively.

The non-destructive FLAV and FLAV_UV indices satisfactorily correlated to the flavonol content of grape berry skin extracts determined by LC-MS analysis as the sum of the quercetin, kaempferol, and isorhamnetin glycosides (Fig 1). Considering all the measurements, a great dispersion in the data was observed with no linear correlation both for FLAV, R^2^ = 0.62 and FLAV_UV, R^2^ = 0.43. Best fitting curves were obtained when the sample batch was split according to different berry development periods: bunch closure-veraison period and berry ripening period. The coefficients of determination R^2^ in pre- and post-veraison periods for FLAV were 0.84 and 0.81 and for FLAV_UV were 0.76 and 0.75, respectively. These results corroborated with previous studies reporting different calibration curves for the flavonol estimation *via* Mx indices; Agati *et al*. [25] reported for the white Vermentino grapes linear fitting curve, with a corresponding R^2^ of 0.76, between the FLAV index and the berry skin flavonol content, while Ferrandino et al. [27] firstly proposed the use of the FLAV_UV index to evaluate the flavonol content in red coloured grapes to avoid the interference effect of anthocyanin on the red excitation of FRF. Then, the use of the FLAV_UV index was investigated in white grapes, finding R^2^ values of the linear correlation higher than 0.80 [27]. Savi and colleagues [28] proposed a curvilinear model to calibrate the FLAV_UV index on five different grape varieties pulled together (R^2^ = 0.35). It is worth noting that each study adopted a different sampling design (timing and procedure) and different chemical analysis protocol to elaborate the fitting curves. Thus, the definition of a standardised methodology to calibrate the instrument on different grape varieties is needful. In this study, for the first time, two different calibration curves were proposed in accordance to the grapevine phenological stages, pre-veraison and ripening periods. The two calibration curves could be differentially used in the two periods for higher properness towards, for example, agronomical treatments during the grape development period and optimal harvest prediction. Moreover, the reliability of the correlation method was proved by UV/Vis analysis of the grape skin extracts. Absorbance of the extracts at 375 nm showed a strong correlation (R^2^ = 0.93) with the sum of flavonol glycosides content obtained with LC-MS analyses for the corresponding samples (S1 Fig). These results confirmed that the choice of the different flavonol compounds, hence the sum of their content, was appropriate for the investigation of the entire absorbance of the extracts at the considered wavelength of 375 nm. Moreover, UV/Vis spectra collected for the extracts showed the absence of significant absorbance in the visible region (400–700 nm), proving that FLAV index, which uses FRF excited at 630 nm as reference signal, is not subjected to systematic error due to interferences originating from extract absorbance in the visible region [40]. Consequently, both the FLAV and FLAV_UV spectroscopic indices can be effectively used for determining the flavonol content of white grape varieties. Due to the better fitting values observed for FLAV, this index was selected to be applied in the following field trials.

### Evaluation of grapes ripening

The ripening of Pinot blanc grapes was evaluated via standard destructive methods and the chlorophyll fluorescence-based method in 2017 and 2018, in eight different vineyards. The 2017 summer was characterised by moderate temperature conditions and frequent rainfalls (473 mm between 1^st^ June and 30^th^ September), the average air temperatures during the berry development period at the valley and hillside vineyards were 22.00 ± 0.33°C and 19.57 ± 0.56°C, respectively. In 2018, the entire production of Te-2 was compromised by a hailstorm that occurred at the blooming period on 21^st^ June, therefore for this experimental site only the 2017 data were taken in consideration. In the last weeks of July 2018, several hot waves resulted in temperatures exceeding 35°C in the valley and over 30°C in the hills; the average rainfall during the berry development period was 308 mm with a diffused grape water stress status. These temperatures influenced the ripening processes, with an earlier berry sugar accumulation in 2018 than in 2017 (Table 1). The berry ripening occurred smoothly with final average TSS values at harvest of 19.23 ± 0.33°Babo in 2017 and 19.61 ± 0.25°Babo in 2018, with a corresponding titratable acidity of 6.14 ± 0.46 g/L and 4.78 ± 0.45 g/L, respectively. The lower average titratable acidity recorded in 2018 in comparison to 2017 was consistent with the warmer climate condition of 2018 vintage [34,41]. The malic acid degradation was generally higher in valley vineyards compared to the hilly ones, and different trends among vintages can be observed. Specifically, the average malic acid content of the berries at harvest time was 3.39 ± 0.19 and 1.73 ± 0.23 g/L in 2017 and 2018, respectively.

**Table 1:** Maturity parameters of Pinot blanc grapes.

Vintage	Site	Pre-harvest			Harvest			
		DOY	Total Soluble Solids (°Babo)	Titratable acidity (g/L)	DOY	Total Soluble Solids (°Babo)	Titratable acidity (g/L)	Malic acid (g/L)
2017	Ep-1	256	19.84 ± 0.12	7.01 ± 0.31	263	20.68 ± 0.08	6.92 ± 0.05	3.87 ± 0.07
	Ep-2		-	-	257	17.57 ± 0.15	7.54 ± 0.33	3.33 ± 0.28
	Na-1	256	18.60 ± 0.69	5.73 ± 0.16	261	18.94 ± 0.09	5.38 ± 0.10	2.50 ± 0.00
	Na-2	256	17.73 ± 0.11	6.28 ± 0.10	270	19.61 ± 0.06	6.69 ± 0.02	3.83 ± 0.03
	Te-1	256	18.92 ± 0.16	4.49 ± 0.06	263	18.70 ± 0.07	3.85 ± 0.01	2,10 ± 0.00
	Te-2	256	18.32 ± 0.24	6.17 ± 0.19	269	19.65 ± 0.04	6.67 ± 0.04	3.23 ± 0.03
	Tr-1	242	18.67 0.04	4.88 ± 0.04	256	19.82 ± 0.15	4.79 ± 0.02	2.95 ± 0.04
	Tr-2	256	18.10 ± 0.11	6.42 ± 0.20	268	18.83 ± 0.18	7.28 ± 0.11	4.00 ± 0.12
					**263**	**19.23 ± 0.33**	**6.14 ± 0.46**	**3.39 ± 0.19**
2018	Ep-1		-	-	254	19.33 ± 0.03	5.92 ± 0.14	2.52 ± 0.19
	Ep-2	254	19.43 ± 0.43	5.40 ± 0.13	256	19.39 ± 0.53	5.37 ± 0.10	1.76 ± 0.16
	Na-1	254	19.08 ± 0.11	3.94 ± 0.04	255	19.35 ± 0.14	3.94 ± 0.04	0.71 ± 0.02
	Na-2	254	18.36 ± 0.16	6.21 ± 0.10	262	18.94 ± 0.17	5.37 ± 0.13	2.10 ± 0.02
	Te-1	254	20.38 ± 0.10	3.11 ± 0.06	254	20.49 ± 0.10	3.11 ± 0.06	1.35 ± 0.06
	Te-2^a^		-	-		-	-	-
	Tr-1	254	20.53 ± 0.17	3.65 ± 0.24	254	20.63 ± 0.18	3.65 ± 0.14	1.46 ± 0.06
	Tr-2	254	18.50 ± 0.22	6.37 ± 0.15	261	19.11 ± 0.09	6.07 ± 0.11	2.21 ± 0.11
					**256**	**19.61 ± 0.25**	**4.78 ± 0.45**	**1.73 ± 0.23**

The mean values ± standard errors, calculated as the pool of three replicates, are reported for the main grape maturity parameters (total soluble solids, titratable acidity, malic acid content) for each vintage and winegrowing location.

^a^Missing values are due to a hailstorm and caused loss of the entire grape production.

**Fig 2:**
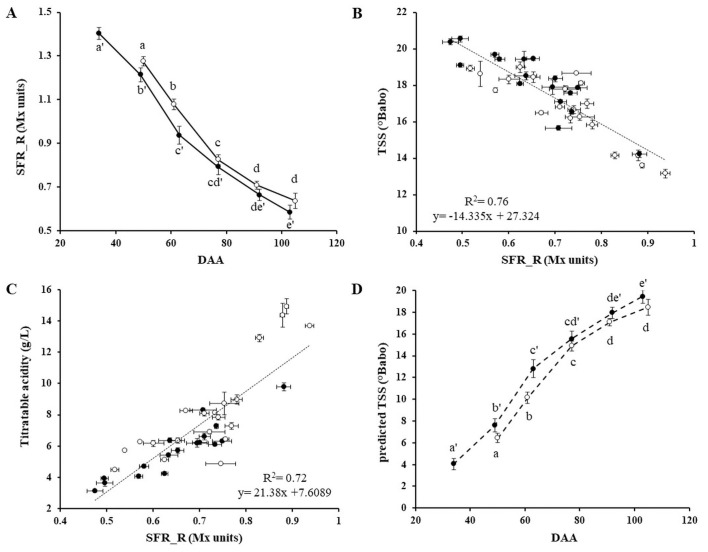
SRF_R index, berry skin chlorophyll content and grape maturity parameters. Evolution of SFR_R index (A) and predicted TSS (D) over Day After Anthesis (DAA) for 2017 (○) and 2018 (●), respectively. Each point represents the mean ± standard errors calculated over the plots under investigation over 6 and 5 time-points during the two vintages. Different letters indicate significant differences according to Tukey’s test (α = 0.05). Correlation between SFR_R (Mx units) and Total Soluble Solids (TSS, °Babo) (B), and between SFR_R and Titratable acidity (g/L) (C). Trend lines, R^2^ coefficient and linear equations are reported in the graphs. Each point represents the average value of three replicates for each variable recorded in 2017 (○) and 2018 (●), respectively. Standard errors of each variable are displayed according to the specific axis position.

The berry-skin chlorophyll content was evaluated *via* non-destructive Mx measurement as SFR_R index (Fig 2). At bunch closure, SFR_R ranged from 1.27 to 1.48 Mx units, with an average value over the plots of 1.40 ± 0.08 Mx units. During ripening, a sharp and continuous decrease in SFR_R was recorded in all the vineyards under investigation, reaching final values close to the harvest time of 0.64 ± 0.04 and 0.58 ± 0.03 Mx units in 2017 and 2018, respectively (Fig 2A). These findings were in accordance with previous studies that proved how chlorophyll degradation is directly associated with berry ripening processes [26,42]. More recently, Balic and colleagues [43] observed in ripening berries of the Thompson Seedless table grape a constant expression of transcripts encoding for chlorophyllase, pheophorbide-a oxygenase, and red chlorophyll catabolite reductase, key role enzymes in the chlorophyll degradation pathway, that can explain the linear chlorophyll breakdown from veraison to grape maturity. On these bases, the SFR_R index can be proposed to noninvasively evaluate the degree of grape ripeness. Indeed, the index well correlated with the berries sugar content of both white and red varieties, suggesting that it can be used as an additional marker of grape ripening [25–27,44,45]. In the present study, a good inverse correlation between SFR_R and TSS in Pinot blanc grapes was observed (R^2^ = 0.76; F(1,35) = 112.757, *p*<0.001) (Fig 2B). Moreover, SFR_R positively correlated with titratable acidity (R^2^ = 0.72; F(1,35) = 90.813, *p*<0.001) (Fig 2C), which naturally decreases during grape ripening [46]. Our analysis showed that SFR_R is suitable to follow the Pinot blanc grape maturity from approximately 14°Babo to more than 20°Babo. By using the linear regression reported in Fig 2B, the sugar concentration in the berry juice starting from the initial ripening phase was estimated from in-field SFR_R measurements. The extrapolated TSS curves (Fig 2D) were in accordance with the dynamic of grape berry growth and physiological ripening previously reported in the literature [47–49]. Indeed, the sugar accumulation rapidly proceeds starting from approximately 60 days after anthesis (DAA) at the onset of veraison [48,49], that occurred around 56 and 61 DAA, in 2017 and 2018, respectively. These results confirmed the reliability of the simple fluorescent ratio (SFR_R) as an indicator of the berry maturity degree in white grapes and can foster its application as a non-destructive technique in viticulture.

### Spatiotemporal dynamics of flavonols in the vineyard under different agro-climatic conditions

**Fig 3:**
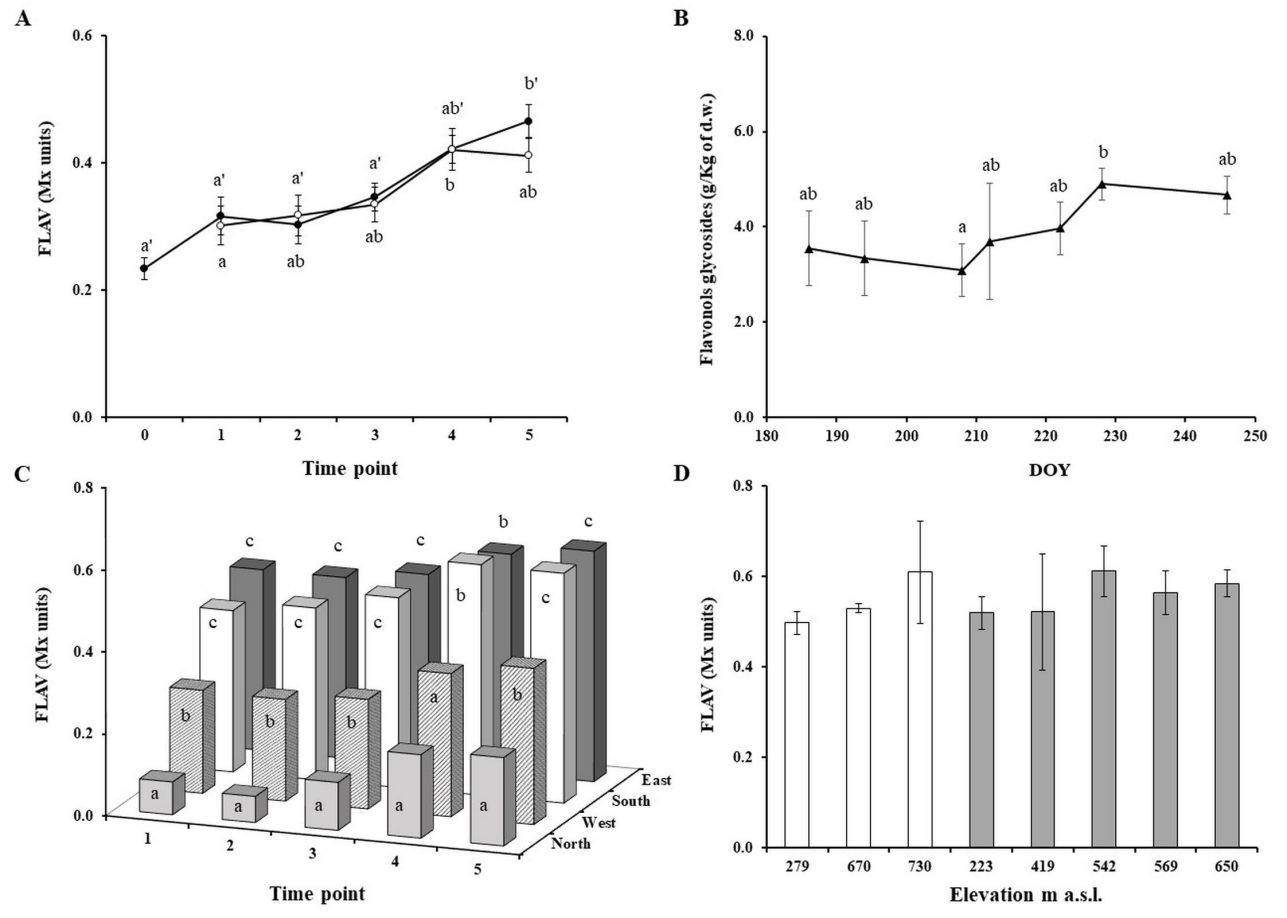
Dynamics of flavonols in Pinot Blanc grape skin during the grape development period and site effect. Trends of FLAV index (Mx units) recorded in the 2017 (○) and 2018 (●) vintages (A). Each point represents the mean ± standard error calculated as the pool of eight and seven vineyards, in 2017 and 2018, respectively at six different time-points (0, DOY185; 1, DOY 200–201; 2, DOY 212–214; 3, DOY 228; 4 DOY 242–243; 5, DOY 254–256). Trend of the total amount of flavonol glycosides extracted from the Pinot blanc skin in Tr-1 (2018) (B). Data are expressed as absolute amounts (g/kg grape skin d.w.). Each point represents the mean ± standard error of six replicates. Site effects on flavonol amount, evaluated as: Grape bunch localisation on the row (C), and site elevation (D). The elevation effect was evaluated *per* group of grape-bunch exposure: South-oriented (white bars), east-oriented bunches (grey bars). Each point represents the FLAV average value calculated as the pool of measurements done in 2017 and 2018 for each time-point (1 to 5 for grape-bunch localisation, and 5 as representative for elevation). Different letters indicate significant differences according to Tukey’s test (*α =* 0.05) among time (A, B) and according to Dunn Bonferroni test (*α* = 0.05) for bunch localisations or elevation (C, D).

The flavonol dynamics in Pinot blanc berry skin was evaluated as FLAV index, due to its best fitting curve with wet chemistry results. The *in vivo* measurements were made during the grape development period, in 2017 and 2018 vintages. At bunch closure (DOY 185; 2018), FLAV in the different locations ranged from 0.15 ± 0.02 and 0.29 ± 0.02 Mx units, with an average value of 0.23 ± 0.02 Mx units. Then, during the berry development period, a general incremental trend in FLAV was observed (Fig 3A). Specifically, the flavonol content remained quite constant until the veraison phase was completed, with an average value of 0.31 Mx units. By middle August, the flavonol content started increasing until reaching an average value of 0.42 Mx units, with a maximum value of 0.62 Mx units recorded in Na-1 (2018). Close to technological maturity, FLAV remained constant or slightly decreased by 5 to 13% of the maximum value recorded in each vineyard, reaching the mean value of 0.41 and 0.47 Mx units at DOY256 and DOY254, in 2017 and 2018, respectively (Fig 3A). It is known that the flavonol synthesis takes place at two key stages of berry development: (i) during the inflorescence development with a peak at flowering, and (ii) starting from veraison when a period of rapid accumulation begins, with an observed peak after 3–4 weeks post-veraison [50,51]. The flavonol dynamics evaluated as spectroscopic index was consistent with the wet chemistry data achieved during the 2018 vintage in Tr-1 (Fig 3B). Indeed, before veraison, the flavonol content ranged from 3.09 ± 0.55 and 3.55 ± 0.77 g/kg of dry weight (d.w.) skin. At veraison, the average value was 3.69 ± 1.22 g/kg d.w. The high variation observed in the data at this time-point could be due to the appreciable asynchrony developmental changes that occur among berries of a grape bunch during this phenological stage [52]. Then, a sharp increase up to 4.90 ± 0.34 g/kg d.w., followed by a final 5% decrement in the flavonol amount, was observed. The flavonol dynamic recorded for Pinot blanc grapes is comparable to previously reported flavonol accumulation curves of Vermentino, Erbaluce, Chardonnay white grapes, even if typical flavonol evolution related to the grape variety has been detected [25,53,54]. Different studies highlighted the role of climatic conditions and sun exposure in determining the evolution of antioxidant compounds and aromatic precursors in ripening grapes [28,55–58]. Considering each vineyard as a specific combination of elevation and row-orientation, these two variables were individually analysed in order to investigate their potential effects on the natural flavonol content of Pinot blanc berry-skins under field condition (Fig 3). The role of the grape bunch-localisation on the row, and the resulting sunlight exposure, appear to be a factor of primary importance for the flavonol accumulation [23,28]. Indeed, taking in consideration that the flavonol biosynthesis is enhanced by sunlight [23], and on the basis of the recorded FLAV values, significant differences were found between the most exposed grape bunches on the E and S row-sides and the less exposed N and W row-sides, at each time-point (Fig 3C). The FLAV values recorded on the E and S row-sides were generally comparable, the lowest flavonol content was instead recorded on the N row-side. The local geomorphology of the valley together with vineyard localisation and row-orientation are determining factors for the penetration of solar radiation in the grape canopy. Hence, we can suppose that, in the Adige-Valley, the W row-side received less global solar radiation compared to the E row-side, as also underlined by the data of the “defoliation trial” (Table 2). On the other hand, the elevation factor, if considered alone, appeared to be irrelevant, indeed no significant differences were found between the different vineyard elevation *per* exposure (Fig 3D).

**Table 2:** Influence of canopy management treatment on the grape-bunch microclimate and berries phenolic content.

Wg area^a^	Bunches row-loc^b^	Leaf removal treatment	Irradiance (W/m^2^)	Inside-bunch temperature (°C)	FLAV (Mx units)	Total polyphenolic content (mg/L)
Eppan	east	ID	144556.24 ± 5526.98 *	22.25 ± 0.10 ^ns^	0.55 ± 0.01*	382.3 ± 5.78 *
		ND	92461.95 ± 14527.39	22.17 ± 0.04	0.44 ± 0.01	342.0 ± 6.81
	west	ID	106589.78 ± 4059.42 *	22.29 ± 0.13 ^ns^	0.39 ± 0.03*	315.3 ± 8.41 ^ns^
		ND	69491.77 ± 9063.14	21.97 ± 0.06	0.25 ± 0.03	332.0 ± 1.15
Terlan	north	ID	95820.54 ± 9741.22 *	24.09 ± 0.02 ^ns^	0.41 ± 0.01 ^ns^	318.7 ± 11.29 *
		ND	70139.59 ± 3128.99	24.28 ± 0.14	0.34 ± 0.02	302.7 ± 10.9
	south	ID	168396.51 ± 4808.18 ^ns^	24.66 ± 0.03 ^ns^	0.61 ± 0.02 ^ns^	385.7 ± 4.26 *
		ND	174644.50 ± 9940.35	24.70 ± 0.15	0.51 ± 0.02	345.3 ± 8.25

The reported microclimate parameters are the average of inside-bunch temperature (°C) and total irradiance (W/m^2^) recorded from DOY 185 to 243. The grape flavonol content and total polyphenolic content of grape juice (mg/L) were measured at DOY 243 by the FLAV index (Mx units) and the Folin-Ciocalteu method, respectively. Each value is the mean ± standard error of three replicates. For each grape row-localisation, asterisks indicate values that significantly differ (Student’s *t*-test; *α* = 0.05) in the pairwise comparison between intense defoliation (ID) and no-defoliation (ND) leaf removal treatments.

^a^Wg area stays for winegrowing area.

^b^Bunches row-loc stays for bunches row-localisation.

**Fig 4:**
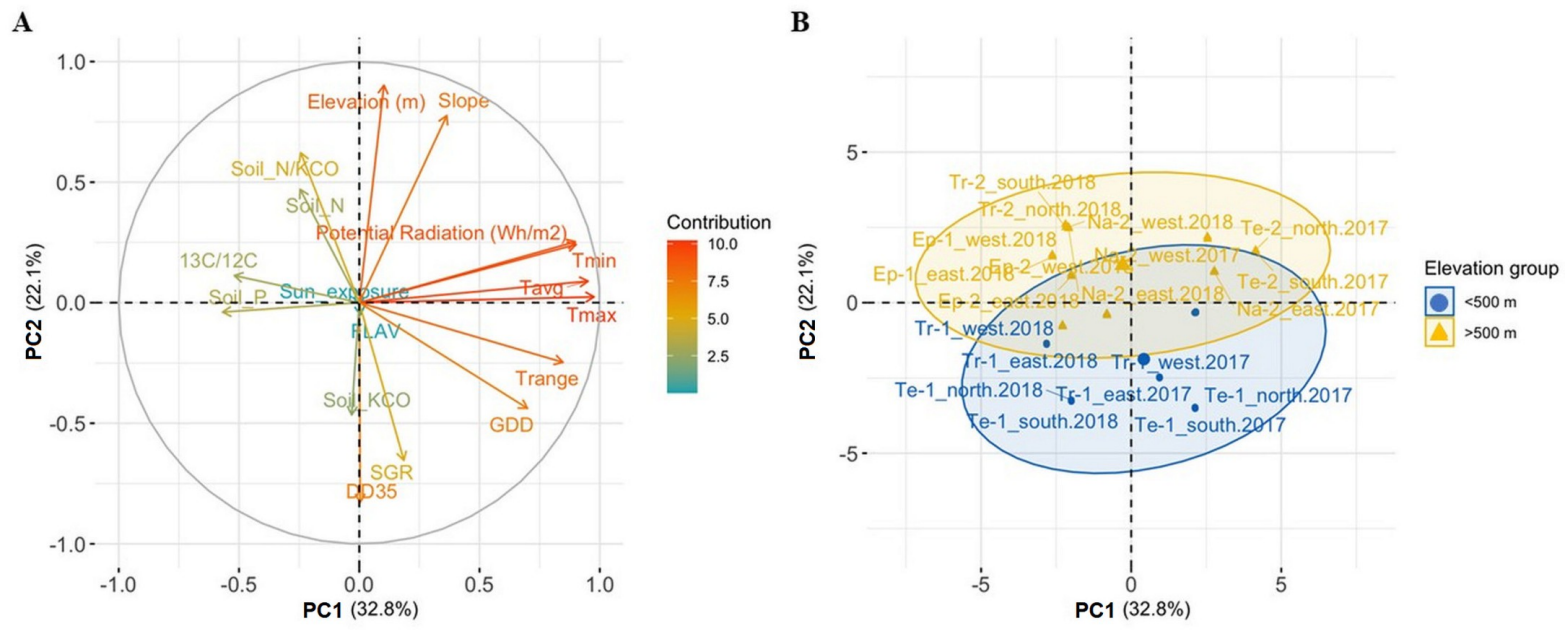
PCA of PC1-PC2 of agro-climatic variables and grape bunch flavonol content, FLAV. Loading plot (A) and scores plot (B) on PC1 and PC2 (observation are grouped according to the elevation range: Green ellipse <500 m a.s.l., blue ellipse >500 m a.s.l.,) showing the contribution of variables and individuals, respectively, to each PC and C) shows the biplot that overlays loadings plot and scores plot on PC1 and PC2. Individual points (coloured by FLAV content averaged on three replicates) represent the observations; variables (arrows) are coloured according to different categories [elevation, temperature parameters and indices, *e*.*g*., GDD and DD35, stable carbon isotopic fraction, slope, and mineral soil content (N, P, and KCO)].

**Fig 5:**
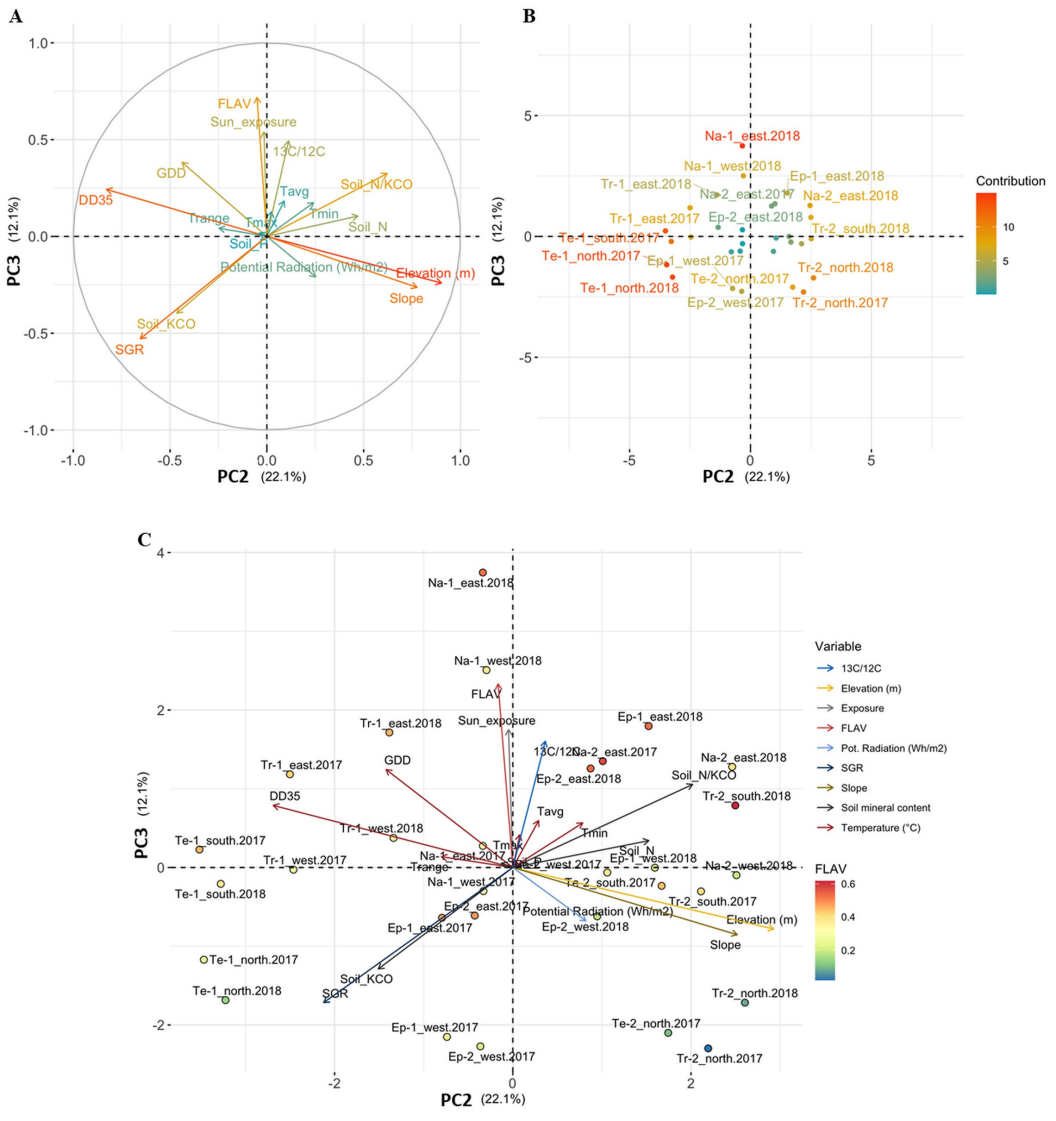
PCA of PC2-PC3 of agro-climatic variables and grape bunch flavonol content, FLAV. Loading plot (A) and scores plot (B) on PC2 and PC3 showing the contribution of variables and individuals, respectively, to each PC, and C) shows the biplot that overlays loadings plot and scores plot on PC2 and PC3. Individual points (coloured by FLAV content averaged on three replicates) represent the observations; variables (arrows) are coloured according to different categories [elevation, temperature parameters and indices, *e*.*g*., GDD and DD35, stable carbon isotopic fraction, slope, and mineral soil content (N, P, and KCO)].

The effect of multiple agro-climatic parameters on grape flavonol content was further evaluated by PCA analysis. For this purpose, the row-sides were classified as “exposed” (E and S) and “covered” (N and W). The first three components cumulatively retained 67.05% of the total variability. The first PC (PC1) was responsible for 32.82% of the variance, the second PC (PC2) accounted for 22.09%, while the third PC (PC3) explained 12.14% of the differences in the data set (S1 and S2 Tables). The climatic parameters strongly contributed to the first two PCs (loadings plot in Fig 4A), and they were able to describe climate features of the sites (scores plot in Fig 4B). Precisely, PC1 correlated mainly with Tmax (r = 0.98) and Tavg (r = 0.95) that differently affect the cooler 2017 and the warmer 2018 vintages. PC2 was associated with field elevation and slope (r = 0.90 and r = 0.78), and it was inversely affected by DD35 (r = -0.83). Moreover, PC2 strongly distinguished between lower sites (below 500 m a.s.l.) characterised by a warmer climate, and upper sites characterised by cooler temperatures and a higher slope of the vineyard site, supporting the findings of Michelini *et al*. [34]. Flavonol content and bunch sunlight-exposure variables were directly associated with PC3 (r = 0.72 and r = 0.54, respectively) (Fig 5). Prior to discussing these results, it has to be considered that anthocyanins and flavonol compounds shared common steps during their biosynthetic process [50], therefore it can be assumed that factors modulating anthocyanin metabolism could also affect the flavonol accumulation (*e*.*g*., water status, temperature, nutrient availability) [59]. The flavonol content of Pinot blanc berry skin (expressed as FLAV and Mx units) seems to be positively influenced by water deficiency measured as ^13^C/^12^C fraction [60,61] (Fig 5A). On the other hand, the controversial role of water availability on grape flavonol content is still an open issue. The flavonol content was reported to be moderately or not consistently affected by drought stress in Cabernet Sauvignon and Merlot [62–65], or even enhanced by irrigation in Tempranillo grapes [59]. Considering the white grape varieties, some key structural flavonoid genes were positively modulated in Tocai Friulano and Chardonnay grapes under water deficit conditions [63,66]. Therefore, further studies are needed to better elucidate the effect of water deficiency on Pinot blanc grape-skin flavonol composition by investigating the specific flavonoid biosynthetic pathways.

Considering the limited information currently available on the effect of nutrient availability and chemical fertilisation on flavonol biosynthesis, our attention focused on the three main soil nutrients (nitrogen, potassium, and phosphate). The PCA analysis showed that the flavonol content of Pinot blanc grapes might not be strongly affected by nitrogen and phosphorus availability and is negatively affected by high potassium concentration. Interestingly, a positive effect of the nitrogen-potassium ratio on flavonol content could be observed (Fig 5A). It has been generally reported that high nitrogen levels delayed the accumulation of phenolic compounds, particularly flavonols, in the grape skin at veraison, although the effect of nitrogen availability generally decreased towards fruit maturity [67]; instead no evidence concerning the effect of both potassium and phosphate on the flavonol content have been so far reported [20,68,69]; even though considering the N x K interaction in Tempranillo grapes, an optimal nutritional nitrogen-potassium ratio might enhance the phenolic features of grape berries [68].

A general statement is that the most likely mechanism for decreasing the polyphenolic content in grape berries is excessive plant vigour [20]. For instance, Sangiovese grapes from high-vigour vines showed a lower flavonol concentration compared to low-vigour plants [70]; in the same way, the flavonol content in Pinot blanc berry skin is negatively affected by plant vigour (SGR) (Fig 5A). Considering the climatic parameters, the flavonol content in grape skin appeared to be slightly affected by the temperature [71], even though by looking at the Tmin variable, it can be assumed that moderate temperature during the berry development period might have enhanced the flavonol content in Pinot blanc berry skin (Fig 5A and 5B). To support our findings, it has been recently reported that low temperature and light have a synergistic effect on the expression of genes in the flavonoid biosynthesis pathway [72]. Despite the previously discussed agro-climatic parameters, sunlight exposure and UV radiation can have a much stronger influence on flavonol biosynthesis [23,73,74]. In the present study, the potential solar radiation variable was unable to discriminate against the sunlight effect because it has been calculated as an average value for the entire plot. Conversely, if the row-side classification as “exposed bunch” (E and S) and “covered bunch” (N and W) is considered, the flavonol content of Pinot blanc berry skin was greater on more-exposed (E and S) than less-exposed (W and N) row-sides (Fig 5C). The observed results were consistent among vintages. Furthermore, based on our observations, in the Adige-Valley, the flavonol accumulation appeared to be generally enhanced by the E exposition of grape bunches to sunlight.

### Flavonol content modulation via leaf removal treatment

**Fig 6:**
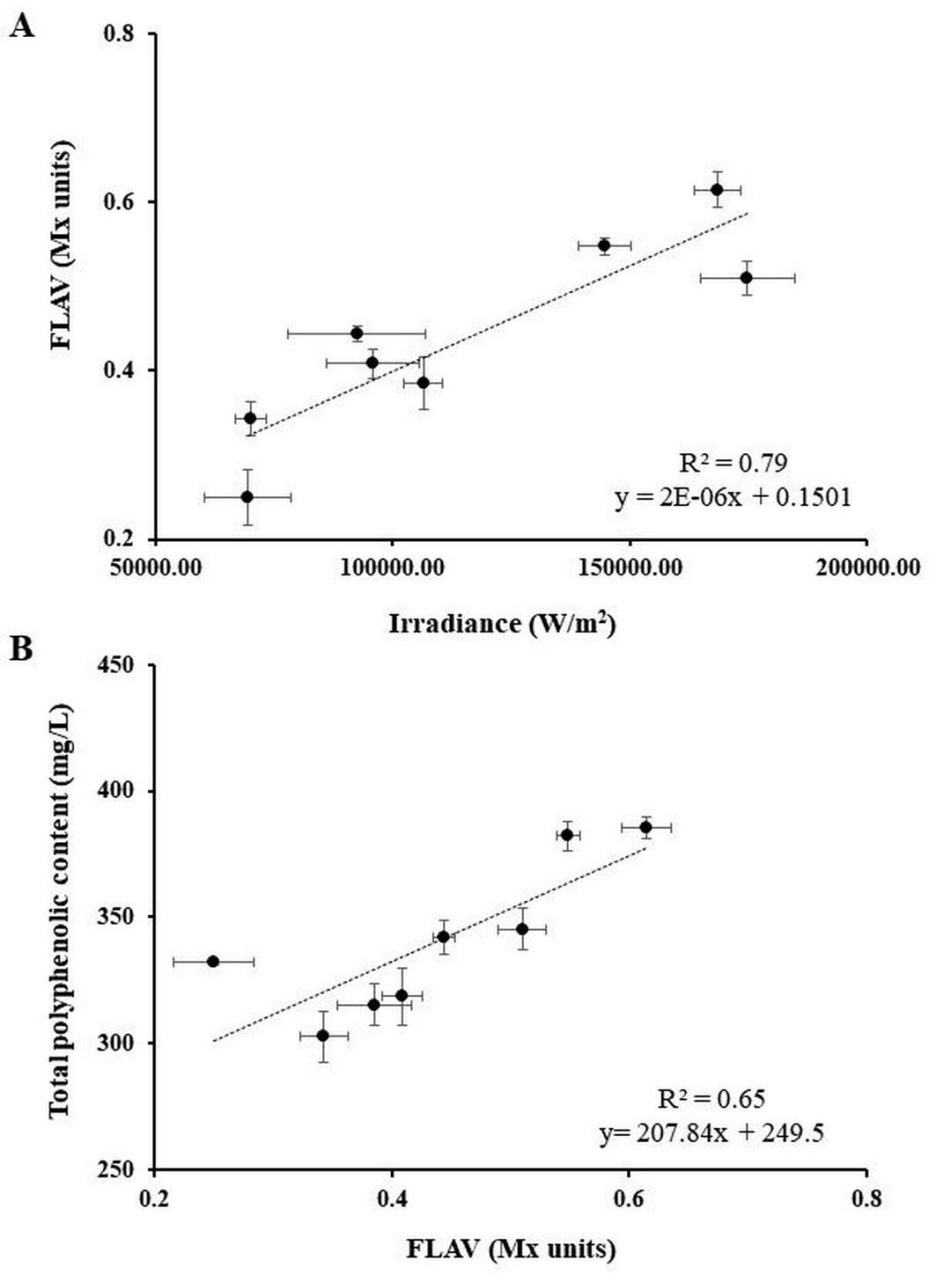
Correlation between FLAV index, environmental factors and grape juice phenolic compounds. Correlation between FLAV (Mx units) at DOY 243 and cumulative irradiance (W/m^2^) recorded in the corresponding bunch-zone during the berry development period (A), and between FLAV and the total polyphenolic content of berry juice at grape ripeness (mg/L) (B). Trend lines, R^2^ coefficient and linear equations are reported in each graph. Each dot represents the average value of three replicates for each variable recorded in the 2018 vintage in Ep-1 and Te-1. Standard errors of each variable are displayed according to the specific axis position.

Considering each grape row-localisation (N, E, S, W), no-significant differences were found between ID and ND treatments regarding the inside-bunch average temperature (Table 2). The global solar radiation affecting the bunch-zone, measured as the sum of irradiance (W/m^2^) recorded hourly from DOY 185 to DOY 243, reached the highest values on the most exposed row-side (S) both in ND and ID treatments, and the lowest values in the ND grapes located on N and W row-sides (Table 2). For each grape row-localisation, the irradiance values of the bunch-zone were significantly higher in ID compared to the ND treatment, except in the case of the S row-side, where a slight non-significant difference between the two treatments was observed (Table 2). A strong linear relationship between the global solar radiation and flavonol content (FLAV index) recorded at the end of the grape ripening period was observed (R^2^ = 0.79; F(1,6) = 22.854, *p* = 0.003) (Fig 6A). Considering the leaf removal effects on the grape skin flavonol accumulation, significant differences in the FLAV index between treatment combinations were maintained along the entire berry development period in the Ep-1 vineyard (S2 Fig). At DOY 243, significant increments in FLAV units of up to 23% and 54% in Ep-E-ID and Ep-W-ID, compared to the corresponding ND treatments, were observed (Table 2). Interestingly, the defoliation treatment made on the W row-side led the grapes to reach the same FLAV value of shaded grapes located on the E row-side, with comparable values recorded along the entire ripening period (S2 Fig). In Terlan, the defoliation treatment applied on the N row-side showed a slight effect on the flavonol accumulation, with no significant differences observed at DOY 243 between Te-N-ID and Te-N-ND for FLAV values. Although the flavonol content of S-located grapes was slightly positively affected by the leaf-removal treatment, FLAV recorded at DOY 243 did not significantly differ between defoliation treatments with 0.61 ± 0.02 and 0.51 ± 0.02 Mx units for Te-S-ID and Te-S-ND, respectively (Table 2). It is worth noting that visual sunburn damages due to overexposure to solar radiation and high temperature (max. inside bunch T above 42° C) were observed on the S-located grapes of both treatments. An abrupt decrease in grape flavonol content was recently reported in severely overexposed Cabernet Sauvignon grapes [75]; therefore, a partial flavonoids degradation effect, rather than down-regulation of their synthesis [76], might potentially explain the lack of differences between Te-S-ID and Te-S-ND.

In the present study, no correlation between the inside-bunch average temperature, recorded during the ripening period, and the final flavonol content was observed (R^2^ = 0.16; F(1,6) = 1.158, *p* = 0.323). A more precise evaluation of the interaction between the two factors (FLAV and temperature) in white grape varieties could be made in the future by measuring the effective grape skin temperature using infra-red thermometers instead of considering the inside-bunch temperature [75]. Although there have been efforts to decouple the interrelationships between temperature and light effects on the flavonoid biosynthesis [71,72,75], this issue is not yet fully elucidated, and hence further field and molecular studies are needed. Overall, the different microclimatic conditions created by the canopy manipulation in the area surrounding the grape bunches can effectively modulate the flavonol content of Pinot blanc berries, as previously reported for red grape varieties [20,21,73].

Several agronomic practices (*e*.*g*., defoliation, application of shadings nets, balancing of the sink/source ratio, thinning of bunches, foliar, and soil fertilisation) have been shown to positively influence primary and secondary metabolites (*i*.*e*., flavonoids) production in white grapes [77]. In the present study, the leaf removal treatment affected the total polyphenolic berry content of grape juice (Table 2). In addition, a good correlation between the FLAV index and the total polyphenolic content of grape juice at harvest was observed (R^2^ = 0.65; F(1,6) = 11.190, *p* = 0.016) (Fig 6B). This result highlighted the possible use of the spectroscopic sensor as a rapid non-destructive tool to evaluate the direct effect of winegrowing practises on the chemical composition of white grapes, and to make possible predictions on the stability and sensory characteristics of the resulting wines [14–17]. Taking into consideration these results, the authors suggest the possible application of the term ‘phenolic maturity’ (that is used for red skinned varieties, so far) to white grapes.

## Conclusion

Since flavonols have a beneficial effect on the storage and antioxidant properties of white wines. Determining their berries skin content and seasonal evolution could represent important information, in addition to the standard oenological parameters, to evaluate the quality of white grapes before harvest. This study provides an exhaustive characterisation of the total flavonol dynamics during the berry development period, using the Pinot blanc variety as an example, and investigates the effects of different agro-climatic parameters (*e*.*g*., temperature, water stress, and irradiance) on flavonol accumulation in the grape skin. Although a weak temperature effect was observed, it is possible to speculate that a moderate temperature during the berry development period may have a positive influence on flavonol accumulation, as well as the water-stress status of the vines. The central role of sunlight-driven accumulation and the specific bunch exposure in modulating the grape skin flavonol content was confirmed. Interestingly, a greater flavonol accumulation was observed on the bunches located on the east and south sides of the vine-rows in the Adige-Valley, despite the fact that the flavonol accumulation on the bunches located in all four cardinal directions could be effectively increased by applying an intense defoliation treatment. These findings emphasise the critical importance of an accurate evaluation of the specific pedoclimatic conditions of the winegrowing region on grape flavonol accumulation, as well as the direct effect of agronomical practices on grape composition. Overall, the present study sheds light on the effective use of non-destructive methods as a decision-making-tool for the prediction of grape quality by: (i) evaluating both the sugar accumulation and titratable acidity reduction as derivate parameters from the chlorophyll content (*e*.*g*., SFR_R index), and (ii) providing an estimation of the flavonol content in pre- and post-veraison and its possible modulation mediated by the application of agronomic practices (*e*.*g*., canopy management treatments).

## Supporting information

S1 Fig Correlation between total flavonol glycosides obtained via LC-MS and the absorbance of the same samples taken at 375 nm. The trend-line of equation y = 0.0548x + 0.0971, R^2^ = 0.93 is displayed in the chart.
(TIF)

S2 Fig Evolution of the FLAV index on Pinot blanc grapes under different canopy management treatments. Accumulation curve of the FLAV index (Mx units) in Ep-1 (A) and Te-1 (B) in the 2018 vintage. Full (●) and empty (○) dot markers indicate the sunlight exposure of grape bunches: W-E in Ep-1, and N–S in Te-1, respectively. Solid-line (─) and dash-line (**‑‐‐**) represent the canopy management treatment of no-defoliation (ND) and intense defoliation (ID), respectively. Each point represents the mean ± standard error calculated as the pool of three replicates for each time-point and treatment. At each time-point, different letters indicate significant differences between treatments according to Tukey’s test (*α* = 0.05).
(TIF)

S1 Table Eigenvalues of the PCA. The table shows the eigenvalues of each component of the PCA as well as the percentage of the total variance which is accounted for by each component and the cumulative percentage accounted by the components.
(DOCX)

S2 Table Eigenvector analysis of PCA. The table shows the eigenvector coefficients of the 17 parameters considered in this study for each component.
(DOCX)

S1 Data (DOCX)
