# Supplementary figures and images for: Non-destructive fluorescence sensing for assessing microclimate, site and defoliation effects on flavonol dynamics and sugar prediction in Pinot blanc grapes
Source: PLoS One. 2022 Aug 16;17(8):e0273166. doi: 10.1371/journal.pone.0273166 (PMC9380915; doi:10.1371/journal.pone.0273166)

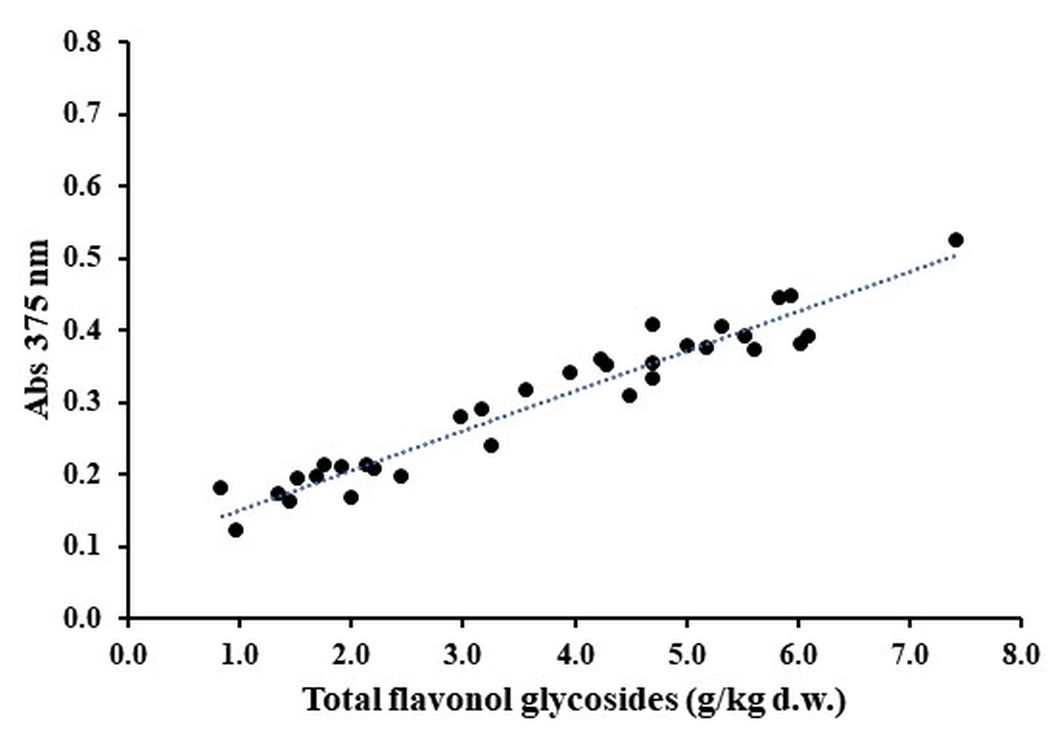

Supplement: S1 Fig — The trend-line of equation y = 0.0548x + 0.0971, R2 = 0.93 is displayed in the chart. (TIF) [file pone.0273166.s001.tif]

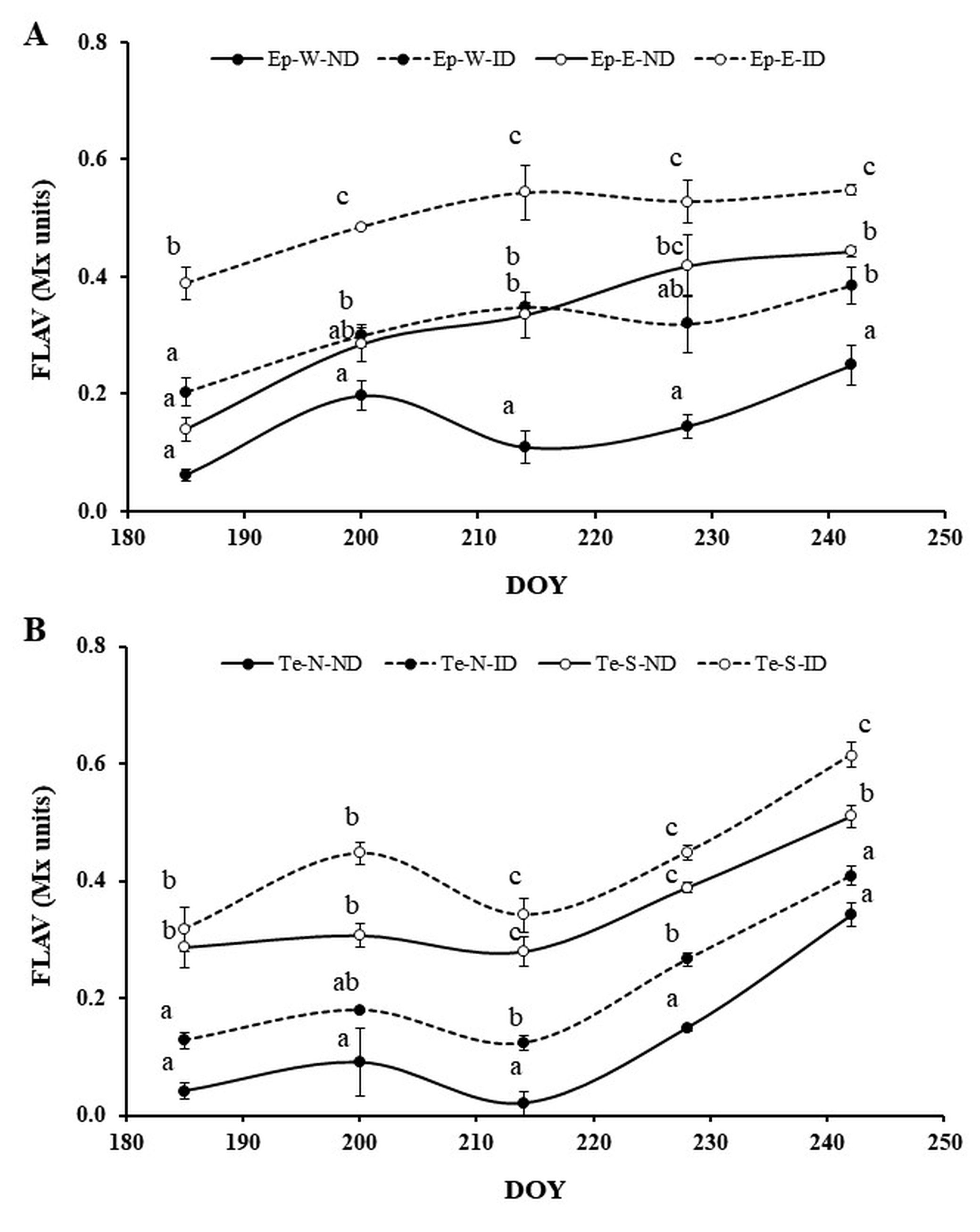

Supplement: S2 Fig — Accumulation curve of the FLAV index (Mx units) in Ep-1 (A) and Te-1 (B) in the 2018 vintage. Full (●) and empty (○) dot markers indicate the sunlight exposure of grape bunches: W-E in Ep-1, and N–S in Te-1, respectively. Solid-line (─) and dash-line (‑‐‐) represent the canopy management treatment of no-defoliation (ND) and intense defoliation (ID), respectively. Each point represents the mean ± standard error calculated as the pool of three replicates for each time-point and treatment. At each time-point, different letters indicate significant differences between treatments according to Tukey’s test (α = 0.05). (TIF) [file pone.0273166.s002.tif]
