# Supplementary material for: Non-destructive fluorescence sensing for assessing microclimate, site and defoliation effects on flavonol dynamics and sugar prediction in Pinot blanc grapes
Source: PLoS One. 2022 Aug 16;17(8):e0273166. doi: 10.1371/journal.pone.0273166 (PMC9380915; doi:10.1371/journal.pone.0273166)
Supplement: S1 Table — The table shows the eigenvalues of each component of the PCA as well as the percentage of the total variance which is accounted for by each component and the cumulative percentage accounted by the components. (DOCX) [file pone.0273166.s003.docx]

| **Dimension** | **Eigenvalue** | **Variance (%)** | **Cumulative variance (%)** |
| --- | --- | --- | --- |
| Dim.1 | 5.579 | 32.816 | 32.816 |
| Dim.2 | 3.755 | 22.089 | 54.905 |
| Dim.3 | 2.064 | 12.140 | 67.045 |
| Dim.4 | 1.682 | 9.897 | 76.942 |
| Dim.5 | 1.549 | 9.110 | 86.052 |
| Dim.6 | 1.181 | 6.947 | 92.999 |
| Dim.7 | 0.508 | 2.986 | 95.985 |
| Dim.8 | 0.235 | 1.382 | 97.368 |
| Dim.9 | 0.192 | 1.128 | 98.496 |
| Dim.10 | 0.104 | 0.613 | 99.109 |
| Dim.11 | 0.068 | 0.398 | 99.507 |
| Dim.12 | 0.052 | 0.304 | 99.810 |
| Dim.13 | 0.026 | 0.154 | 99.964 |
| Dim.14 | 0.005 | 0.029 | 99.994 |
| Dim.15 | 0.001 | 0.004 | 99.998 |
| Dim.16 | 0.000 | 0.002 | 100.000 |
| Dim.17 | 0.000 | 0.000 | 100.000 |

**S1 Table. Eigenvalues of the PCA.** The table shows the eigenvalues of each component of the PCA as well as the percentage of the total variance which is accounted for by each component and the cumulative percentage accounted by the components.
