# Supplementary material for: Non-destructive fluorescence sensing for assessing microclimate, site and defoliation effects on flavonol dynamics and sugar prediction in Pinot blanc grapes
Source: PLoS One. 2022 Aug 16;17(8):e0273166. doi: 10.1371/journal.pone.0273166 (PMC9380915; doi:10.1371/journal.pone.0273166)
Supplement: S2 Table — The table shows the eigenvector coefficients of the 17 parameters considered in this study for each component. (DOCX) [file pone.0273166.s004.docx]

**S2 Table. Eigenvector analysis of PCA.** The table shows the eigenvector coefficients of the 17 parameters considered in this study for each component.

|  | **PC1** | **PC2** | **PC3** | **PC4** | **PC5** | **PC6** | **PC7** | **PC8** | **PC9** | **PC10** | **PC11** | **PC12** |
| --- | --- | --- | --- | --- | --- | --- | --- | --- | --- | --- | --- | --- |
| **Elevation (m)** | -0.0435 | -0.4655 | 0.1677 | 0.1804 | -0.0360 | 0.1326 | -0.0364 | 0.3181 | 0.0561 | 0.2070 | 0.2126 | -0.2912 |
| **Sun_exposure** | -0.0004 | 0.0076 | -0.3755 | 0.5273 | -0.2955 | -0.0822 | 0.2943 | -0.3267 | -0.0207 | 0.5080 | 0.0614 | 0.1683 |
| **Slope** | -0.1541 | -0.4004 | 0.1832 | 0.1101 | -0.0669 | 0.1626 | 0.4225 | 0.1895 | -0.3194 | -0.2424 | 0.3269 | 0.2826 |
| **FLAV** | -0.0020 | 0.0263 | -0.4999 | 0.4504 | -0.2030 | -0.0186 | -0.1814 | 0.3128 | 0.0209 | -0.5695 | -0.0716 | -0.1998 |
| **Tavg** | -0.4029 | -0.0461 | -0.1276 | -0.1132 | -0.0454 | 0.1093 | -0.1287 | -0.1177 | -0.0024 | 0.0322 | -0.0304 | 0.0505 |
| **Tmin** | -0.3819 | -0.1248 | -0.1215 | -0.0583 | 0.0416 | 0.2434 | -0.0637 | -0.2129 | 0.0561 | -0.0756 | 0.1732 | -0.0563 |
| **Tmax** | -0.4134 | -0.0124 | -0.0890 | -0.0908 | -0.0614 | 0.0124 | -0.0982 | 0.0870 | 0.0401 | 0.1181 | 0.0598 | -0.0977 |
| **Trange** | -0.3587 | 0.1275 | -0.0294 | -0.1101 | -0.1732 | -0.2722 | -0.1181 | 0.4333 | 0.0116 | 0.3278 | -0.0920 | -0.1262 |
| **GDD** | -0.2962 | 0.2261 | -0.2667 | -0.2336 | 0.0094 | 0.2030 | 0.0287 | -0.2712 | -0.0745 | -0.1690 | 0.3005 | 0.0046 |
| **DD35** | -0.0016 | 0.4275 | -0.1691 | -0.1877 | 0.0609 | -0.1796 | 0.4302 | 0.4465 | -0.0974 | 0.0044 | 0.2301 | 0.2088 |
| **Potential Radiation**  **(Wh/m2)** | -0.3805 | -0.1304 | 0.1455 | 0.0315 | -0.1592 | -0.0872 | 0.0770 | 0.0576 | -0.1770 | -0.0065 | -0.5430 | 0.1931 |
| **Soil_N** | 0.1041 | -0.2428 | -0.0732 | -0.3644 | -0.4805 | -0.2635 | 0.1839 | -0.1988 | -0.0217 | -0.2760 | -0.2447 | 0.0717 |
| **Soil_P** | 0.2406 | 0.0203 | -0.0120 | -0.1611 | -0.5107 | 0.2327 | -0.5233 | 0.1576 | -0.1393 | 0.1443 | 0.2439 | 0.4122 |
| **Soil_KCO** | 0.0134 | 0.2401 | 0.2764 | -0.0595 | -0.5308 | 0.2755 | 0.3318 | -0.0104 | 0.3718 | -0.0275 | 0.0694 | -0.4133 |
| **Soil_N/KCO** | 0.1031 | -0.3208 | -0.2263 | -0.2708 | -0.0392 | -0.5020 | -0.0206 | -0.1040 | -0.1451 | 0.0802 | 0.3370 | -0.3590 |
| **SGR** | -0.0785 | 0.3374 | 0.3680 | 0.2311 | -0.1437 | -0.1457 | -0.1399 | -0.1931 | -0.6699 | -0.0805 | 0.1028 | -0.2910 |
| **13C/12C** | 0.2200 | -0.0580 | -0.3437 | -0.2409 | 0.0581 | 0.4997 | 0.1637 | 0.1227 | -0.4635 | 0.2203 | -0.3312 | -0.3092 |

|  | **PC13** | **PC14** | **PC15** | **PC16** | **PC17** |
| --- | --- | --- | --- | --- | --- |
| **Elevation (m)** | 0.3311 | 0.1322 | -0.5177 | -0.1682 | 0.0000 |
| **Sun_exposure** | 0.0340 | 0.0404 | -0.0057 | 0.0079 | 0.0000 |
| **Slope** | -0.2289 | 0.0948 | 0.3383 | -0.0401 | 0.0000 |
| **FLAV** | -0.0415 | -0.0503 | 0.0072 | -0.0099 | 0.0000 |
| **Tavg** | 0.1928 | -0.3900 | 0.1945 | -0.7282 | 0.0000 |
| **Tmin** | 0.3174 | -0.2174 | 0.0705 | 0.5338 | -0.4797 |
| **Tmax** | 0.1237 | 0.0646 | 0.1792 | 0.3101 | 0.7843 |
| **Trange** | -0.1405 | 0.3940 | 0.2714 | -0.0327 | -0.3933 |
| **GDD** | -0.2947 | 0.4301 | -0.4494 | -0.1466 | 0.0000 |
| **DD35** | 0.3174 | -0.2766 | -0.2206 | 0.0324 | 0.0000 |
| **Potential Radiation**  **(Wh/m2)** | -0.3090 | -0.2961 | -0.4508 | 0.1550 | 0.0000 |
| **Soil_N** | 0.4335 | 0.2970 | -0.0028 | -0.0247 | 0.0000 |
| **Soil_P** | -0.0673 | -0.1284 | -0.0625 | 0.0861 | 0.0000 |
| **Soil_KCO** | -0.1928 | -0.2005 | 0.0482 | -0.0091 | 0.0000 |
| **Soil_N/KCO** | -0.3281 | -0.3414 | -0.0311 | 0.0453 | 0.0000 |
| **SGR** | 0.2043 | 0.0054 | 0.0030 | -0.0092 | 0.0000 |
| **13C/12C** | -0.0096 | 0.0028 | 0.0884 | 0.0254 | 0.0000 |
