# Supplementary material for: Non-destructive fluorescence sensing for assessing microclimate, site and defoliation effects on flavonol dynamics and sugar prediction in Pinot blanc grapes
Source: PLoS One. 2022 Aug 16;17(8):e0273166. doi: 10.1371/journal.pone.0273166 (PMC9380915; doi:10.1371/journal.pone.0273166)
Supplement: S1 Data — (DOCX) [file pone.0273166.s005.docx]

| Fig1 & S1_Fig Calibration curve data | | | | | |
| --- | --- | --- | --- | --- | --- |
| sample | period | FLAV | FLAV_UV | Flavonol (g/kg of d.w.) | Absorbance 375 nm |
| *1* | bunch closure-veraison | 0.37 | 0.40 | 6.106 | 0.392 |
| *2* | bunch closure-veraison | -0.06 | -0.20 | 2.454 | 0.196 |
| *3* | bunch closure-veraison | 0.00 | -0.16 | 2.001 | 0.167 |
| *4* | bunch closure-veraison | 0.18 | 0.03 | 6.029 | 0.380 |
| *5* | bunch closure-veraison | -0.04 | -0.21 | 1.454 | 0.162 |
| *6* | bunch closure-veraison | -0.02 | -0.21 | 1.925 | 0.209 |
| *7* | bunch closure-veraison | -0.09 | -0.25 | 0.968 | 0.121 |
| *8* | bunch closure-veraison | 0.34 | 0.38 | 5.321 | 0.403 |
| *9* | bunch closure-veraison | -0.06 | -0.19 | 1.515 | 0.193 |
| *10* | bunch closure-veraison | 0.02 | -0.11 | 3.178 | 0.290 |
| *11* | bunch closure-veraison | 0.17 | 0.12 | 5.195 | 0.376 |
| *12* | bunch closure-veraison | 0.28 | 0.36 | 4.297 | 0.352 |
| *13* | bunch closure-veraison | -0.08 | -0.21 | 1.692 | 0.196 |
| *14* | bunch closure-veraison | 0.12 | 0.16 | 2.978 | 0.279 |
| *15* | bunch closure-veraison | 0.29 | 0.32 | 3.574 | 0.317 |
| *16* | bunch closure-veraison | 0.32 | 0.54 | 5.950 | 0.447 |
| *17* | bunch closure-veraison | 0.50 | 0.77 | 7.419 | 0.525 |
| *18* | berry ripening | -0.07 | -0.17 | 1.350 | 0.172 |
| *19* | berry ripening | 0.05 | 0.03 | 1.770 | 0.212 |
| *20* | berry ripening | -0.07 | -0.05 | 2.148 | 0.213 |
| *21* | berry ripening | 0.10 | 0.26 | 2.206 | 0.206 |
| *22* | berry ripening | 0.21 | 0.51 | 4.698 | 0.353 |
| *23* | berry ripening | 0.37 | 0.74 | 3.253 | 0.239 |
| *24* | berry ripening | 0.36 | 0.65 | 4.504 | 0.309 |
| *25* | berry ripening | 0.75 | 1.40 | 5.522 | 0.390 |
| *26* | berry ripening | 0.66 | 1.31 | 5.609 | 0.372 |
| *27* | berry ripening | 0.54 | 1.06 | 4.706 | 0.333 |
| *28* | berry ripening | 0.78 | 1.54 | 5.010 | 0.379 |
| *29* | berry ripening | 0.63 | 1.50 | 5.840 | 0.444 |
| *30* | berry ripening | 0.42 | 1.06 | 4.244 | 0.360 |
| *31* | berry ripening | -0.02 | 0.27 | 0.835 | 0.180 |
| *32* | berry ripening | 0.49 | 1.45 | 3.958 | 0.340 |
| *33* | berry ripening | 0.67 | 1.21 | 4.702 | 0.408 |

Fig.2A

| 2017 | CHL0 | CHL1 | CHL2 | CHL3 | CHL4 | CHL5 |
| --- | --- | --- | --- | --- | --- | --- |
| DOY | 185 | 201 | 212 | 228 | 242 | 256 |
| average | na | 1.275 | 1.079 | 0.825 | 0.709 | 0.636 |
| er.st | na | 0.022 | 0.025 | 0.023 | 0.018 | 0.036 |
| 2018 | CHL0 | CHL1 | CHL2 | CHL3 | CHL4 | CHL5 |
| DOY | 185 | 200 | 214 | 228 | 243 | 254 |
| average | 1.403 | 1.214 | 0.937 | 0.792 | 0.664 | 0.584 |
| er.st | 0.026 | 0.031 | 0.040 | 0.035 | 0.025 | 0.032 |

Fig.2D

| 2017 | Predicted TSS °Babo | | | | | |
| --- | --- | --- | --- | --- | --- | --- |
| DOY | 185 | 201 | 212 | 228 | 242 | 256 |
| average | na | 6.437 | 10.138 | 14.906 | 17.083 | 18.454 |
| er.st | na | 0.446 | 0.504 | 0.464 | 0.363 | 0.729 |
| 2018 | Predicted TSS °Babo | | | | | |
| DOY | 185 | 200 | 214 | 228 | 243 | 254 |
| average | 4.039 | 7.599 | 12.808 | 15.528 | 17.936 | 19.433 |
| er.st | 0.537 | 0.625 | 0.813 | 0.701 | 0.504 | 0.608 |

Fig.2B-C

|  | SFR_R | BABO | Titratable acidity g/L | er.st SFR | er.st Babo | er.st TA |
| --- | --- | --- | --- | --- | --- | --- |
| 2017 | 0.769 | 16.978 | 7.294 | 0.015 | 0.256 | 0.219 |
| 2017 | 0.746 | 18.665 | 4.880 | 0.032 | 0.037 | 0.040 |
| 2017 | 0.937 | 13.150 | 13.687 | 0.010 | 0.248 | 0.041 |
| 2017 | 0.733 | 16.187 | 8.133 | 0.001 | 0.244 | 0.078 |
| 2017 | 0.757 | 18.100 | 6.423 | 0.006 | 0.106 | 0.070 |
| 2017 | 0.888 | 13.590 | 14.918 | 0.004 | 0.135 | 0.481 |
| 2017 | 0.879 | 14.143 | 14.353 | 0.005 | 0.290 | 0.769 |
| 2017 | 0.754 | 16.260 | 8.727 | 0.031 | 0.200 | 0.731 |
| 2017 | 0.721 | 17.790 | 6.910 | 0.033 | 0.165 | 0.127 |
| 2017 | 0.780 | 15.837 | 8.976 | 0.012 | 0.226 | 0.285 |
| 2017 | 0.653 | 18.427 | 6.353 | 0.014 | 0.294 | 0.155 |
| 2017 | 0.538 | 18.603 | 5.727 | 0.002 | 0.690 | 0.063 |
| 2017 | 0.671 | 16.487 | 8.247 | 0.014 | 0.081 | 0.049 |
| 2017 | 0.572 | 17.727 | 6.280 | 0.002 | 0.115 | 0.049 |
| 2017 | 0.741 | 16.655 | 7.843 | 0.014 | 0.224 | 0.168 |
| 2017 | 0.625 | 18.980 | 5.133 | 0.009 | 0.290 | 0.020 |
| 2017 | 0.517 | 18.920 | 4.490 | 0.008 | 0.157 | 0.072 |
| 2017 | 0.829 | 14.153 | 12.897 | 0.009 | 0.179 | 0.246 |
| 2017 | 0.710 | 16.807 | 8.103 | 0.010 | 0.053 | 0.176 |
| 2017 | 0.601 | 18.320 | 6.167 | 0.022 | 0.235 | 0.187 |
| 2018 | 0.749 | 17.865 | 6.315 | 0.021 | 0.083 | 0.114 |
| 2018 | 0.581 | 19.421 | 4.707 | 0.010 | 0.099 | 0.102 |
| 2018 | 0.495 | 20.530 | 3.650 | 0.018 | 0.170 | 0.244 |
| 2018 | 0.736 | 16.565 | 7.269 | 0.005 | 0.157 | 0.153 |
| 2018 | 0.637 | 18.500 | 6.367 | 0.024 | 0.220 | 0.149 |
| 2018 | 0.881 | 14.206 | 9.797 | 0.016 | 0.161 | 0.255 |
| 2018 | 0.733 | 17.558 | 6.102 | 0.014 | 0.099 | 0.113 |
| 2018 | 0.653 | 19.440 | 5.710 | 0.014 | 0.080 | 0.167 |
| 2018 | 0.695 | 17.890 | 6.196 | 0.024 | 0.393 | 0.262 |
| 2018 | 0.634 | 19.430 | 5.403 | 0.020 | 0.430 | 0.131 |
| 2018 | 0.625 | 18.084 | 4.248 | 0.007 | 0.099 | 0.104 |
| 2018 | 0.495 | 19.080 | 3.943 | 0.008 | 0.110 | 0.045 |
| 2018 | 0.707 | 15.652 | 8.316 | 0.029 | 0.137 | 0.116 |
| 2018 | 0.701 | 18.360 | 6.207 | 0.015 | 0.160 | 0.095 |
| 2018 | 0.711 | 17.108 | 6.602 | 0.013 | 0.095 | 0.201 |
| 2018 | 0.570 | 19.668 | 4.065 | 0.009 | 0.128 | 0.128 |
| 2018 | 0.474 | 20.380 | 3.113 | 0.017 | 0.170 | 0.057 |

| DOY | Flavonol (g/kg of d.w.) | |
| --- | --- | --- |
|  | mean | st.er |
| 186 | 3.550347 | 0.77421928 |
| 194 | 3.339842 | 0.77734065 |
| 208 | 3.089621 | 0.54654705 |
| 212 | 3.691907 | 1.22290188 |
| 222 | 3.975048 | 0.55636048 |
| 228 | 4.896834 | 0.33514834 |
| 246 | 4.67030 | 0.40209637 |

Flavonol dynamics Fig3

| 2017 | FLAV_0 | FLAV_1 | FLAV_2 | FLAV_3 | FLAV_4 | FLAV_5 |
| --- | --- | --- | --- | --- | --- | --- |
| DOY | 185 | 201 | 212 | 228 | 242 | 256 |
| average |  | 0.31 | 0.31 | 0.34 | 0.43 | 0.42 |
| st.er |  | 0.02 | 0.03 | 0.03 | 0.02 | 0.02 |
| 2018 | FLAV_0 | FLAV_1 | FLAV_2 | FLAV_3 | FLAV_4 | FLAV_5 |
| DOY | 185 | 200 | 214 | 228 | 243 | 254 |
| average | 0.24 | 0.31 | 0.32 | 0.34 | 0.43 | 0.44 |
| er.st | 0.02 | 0.03 | 0.03 | 0.02 | 0.02 | 0.02 |

| Field | Year | altitude_msl | sun_exposure | FLAV_0 | FLAV_1 | FLAV_2 | FLAV_3 | FLAV_4 | FLAV_5 |
| --- | --- | --- | --- | --- | --- | --- | --- | --- | --- |
| Tr_U | 2017 | 223 | east |  | 0.37 | 0.38 | 0.44 | 0.49 |  |
| Tr_U | 2017 | 223 | east |  | 0.49 | 0.44 | 0.42 | 0.58 |  |
| Tr_U | 2017 | 223 | east |  | 0.47 | 0.35 | 0.41 | 0.59 |  |
| Tr_U | 2018 | 223 | east | 0.36 | 0.47 | 0.36 | 0.42 | 0.49 | 0.53 |
| Tr_U | 2018 | 223 | east | 0.36 | 0.52 | 0.47 | 0.46 | 0.51 | 0.48 |
| Tr_U | 2018 | 223 | east | 0.38 | 0.46 | 0.42 | 0.48 | 0.49 | 0.55 |
| Tr_U | 2017 | 223 | west |  | 0.28 | 0.26 | 0.27 | 0.25 |  |
| Tr_U | 2017 | 223 | west |  | 0.32 | 0.27 | 0.33 | 0.38 |  |
| Tr_U | 2017 | 223 | west |  | 0.29 | 0.26 | 0.24 | 0.40 |  |
| Tr_U | 2018 | 223 | west | 0.20 | 0.19 | 0.22 | 0.31 | 0.24 | 0.34 |
| Tr_U | 2018 | 223 | west | 0.08 | 0.20 | 0.22 | 0.24 | 0.24 | 0.22 |
| Tr_U | 2018 | 223 | west | 0.26 | 0.31 | 0.23 | 0.27 | 0.30 | 0.29 |
| Tr_O | 2017 | 730 | north |  | 0.02 | -0.01 | 0.05 | 0.09 | 0.01 |
| Tr_O | 2017 | 730 | north |  | -0.08 | -0.01 | -0.04 | 0.04 | 0.06 |
| Tr_O | 2017 | 730 | north |  | -0.04 | 0.01 | 0.04 | 0.08 | 0.08 |
| Tr_O | 2018 | 730 | north | 0.04 | 0.04 | 0.07 | 0.09 | 0.07 | 0.17 |
| Tr_O | 2018 | 730 | north | 0.06 | 0.08 | 0.05 | 0.06 | 0.13 | 0.26 |
| Tr_O | 2018 | 730 | north | 0.04 | 0.12 | 0.11 | 0.08 | 0.12 | 0.25 |
| Tr_O | 2017 | 730 | south |  | 0.39 | 0.45 | 0.38 | 0.45 | 0.49 |
| Tr_O | 2017 | 730 | south |  | 0.35 | 0.53 | 0.43 | 0.56 | 0.49 |
| Tr_O | 2017 | 730 | south |  | 0.31 | 0.55 | 0.39 | 0.56 | 0.56 |
| Tr_O | 2018 | 730 | south | 0.44 | 0.54 | 0.59 | 0.63 | 0.61 | 0.65 |
| Tr_O | 2018 | 730 | south | 0.43 | 0.50 | 0.69 | 0.61 | 0.65 | 0.73 |
| Tr_O | 2018 | 730 | south | 0.35 | 0.63 | 0.62 | 0.61 | 0.66 | 0.74 |
| Ep_U | 2017 | 542 | east |  | 0.40 | 0.48 | 0.50 | 0.60 | 0.53 |
| Ep_U | 2017 | 542 | east |  | 0.42 | 0.42 | 0.44 | 0.60 | 0.56 |
| Ep_U | 2017 | 542 | east |  | 0.38 | 0.40 | 0.48 | 0.60 | 0.62 |
| Ep_U | 2018 | 542 | east | 0.31 | 0.51 | 0.52 | 0.53 | 0.58 | 0.65 |
| Ep_U | 2018 | 542 | east | 0.34 | 0.47 | 0.49 | 0.52 | 0.56 | 0.68 |
| Ep_U | 2018 | 542 | east | 0.30 | 0.38 | 0.41 | 0.54 | 0.44 | 0.63 |
| Ep_U | 2017 | 542 | west |  | 0.24 | 0.27 | 0.26 | 0.43 | 0.38 |
| Ep_U | 2017 | 542 | west |  | 0.24 | 0.19 | 0.24 | 0.47 | 0.42 |
| Ep_U | 2017 | 542 | west |  | 0.18 | 0.14 | 0.22 | 0.38 | 0.43 |
| Ep_U | 2018 | 542 | west | 0.09 | 0.20 | 0.20 | 0.22 | 0.31 | 0.47 |
| Ep_U | 2018 | 542 | west | 0.12 | 0.23 | 0.19 | 0.16 | 0.30 | 0.48 |
| Ep_U | 2018 | 542 | west | 0.10 | 0.23 | 0.21 | 0.24 | 0.28 | 0.41 |
| Ep_O | 2017 | 569 | east |  | 0.42 | 0.42 | 0.42 | 0.48 | 0.52 |
| Ep_O | 2017 | 569 | east |  | 0.47 | 0.49 | 0.47 | 0.58 | 0.50 |
| Ep_O | 2017 | 569 | east |  | 0.46 | 0.46 | 0.56 | 0.59 | 0.55 |
| Ep_O | 2018 | 569 | east | 0.26 | 0.42 | 0.37 | 0.45 | 0.43 | 0.59 |
| Ep_O | 2018 | 569 | east | 0.25 | 0.44 | 0.46 | 0.54 | 0.58 | 0.61 |
| Ep_O | 2018 | 569 | east | 0.27 | 0.50 | 0.45 | 0.61 | 0.58 | 0.62 |
| Ep_O | 2017 | 569 | west |  | 0.14 | 0.12 | 0.11 | 0.25 | 0.25 |
| Ep_O | 2017 | 569 | west |  | 0.16 | 0.25 | 0.25 | 0.37 | 0.33 |
| Ep_O | 2017 | 569 | west |  | 0.17 | 0.24 | 0.26 | 0.39 | 0.35 |
| Ep_O | 2018 | 569 | west | 0.17 | 0.19 | 0.18 | 0.16 | 0.23 | 0.25 |
| Ep_O | 2018 | 569 | west | 0.18 | 0.26 | 0.27 | 0.15 | 0.21 | 0.40 |
| Ep_O | 2018 | 569 | west | 0.21 | 0.25 | 0.23 | 0.25 | 0.34 | 0.35 |
| Te_U | 2017 | 279 | north |  | 0.13 | 0.15 | 0.29 | 0.29 | 0.32 |
| Te_U | 2017 | 279 | north |  | 0.12 | 0.16 | 0.24 | 0.33 | 0.30 |
| Te_U | 2017 | 279 | north |  | 0.16 | 0.18 | 0.24 | 0.30 | 0.33 |
| Te_U | 2018 | 279 | north | 0.06 | 0.11 | 0.00 | 0.16 | 0.28 | 0.26 |
| Te_U | 2018 | 279 | north | 0.03 | 0.04 | -0.04 | 0.12 | 0.32 | 0.22 |
| Te_U | 2018 | 279 | north | 0.05 | 0.09 | 0.01 | 0.14 | 0.34 | 0.24 |
| Te_U | 2017 | 279 | south |  | 0.28 | 0.30 | 0.50 | 0.55 | 0.46 |
| Te_U | 2017 | 279 | south |  | 0.32 | 0.28 | 0.39 | 0.44 | 0.50 |
| Te_U | 2017 | 279 | south |  | 0.37 | 0.33 | 0.48 | 0.49 | 0.53 |
| Te_U | 2018 | 279 | south | 0.28 | 0.37 | 0.22 | 0.36 | 0.58 | 0.49 |
| Te_U | 2018 | 279 | south | 0.19 | 0.23 | 0.27 | 0.42 | 0.48 | 0.49 |
| Te_U | 2018 | 279 | south | 0.26 | 0.26 | 0.24 | 0.31 | 0.57 | 0.52 |
| Te_O | 2017 | 670 | north |  | 0.16 | 0.09 | 0.12 | 0.24 | 0.26 |
| Te_O | 2017 | 670 | north |  | 0.12 | 0.16 | 0.05 | 0.17 | 0.19 |
| Te_O | 2017 | 670 | north |  | 0.13 | 0.13 | 0.13 | 0.23 | 0.22 |
| Te_O | 2018 | 670 | north |  |  |  |  |  |  |
| Te_O | 2018 | 670 | north |  |  |  |  |  |  |
| Te_O | 2018 | 670 | north |  |  |  |  |  |  |
| Te_O | 2017 | 670 | south |  | 0.42 | 0.35 | 0.44 | 0.60 | 0.54 |
| Te_O | 2017 | 670 | south |  | 0.38 | 0.41 | 0.42 | 0.56 | 0.52 |
| Te_O | 2017 | 670 | south |  | 0.45 | 0.40 | 0.48 | 0.62 | 0.53 |
| Te_O | 2018 | 670 | south |  |  |  |  |  |  |
| Te_O | 2018 | 670 | south |  |  |  |  |  |  |
| Te_O | 2018 | 670 | south |  |  |  |  |  |  |
| Na_U | 2017 | 419 | west |  | 0.28 | 0.30 | 0.31 | 0.54 | 0.42 |
| Na_U | 2017 | 419 | west |  | 0.25 | 0.29 | 0.38 | 0.39 | 0.42 |
| Na_U | 2017 | 419 | west |  | 0.32 | 0.29 | 0.32 | 0.39 | 0.42 |
| Na_U | 2018 | 419 | west | 0.30 | 0.44 | 0.27 | 0.35 | 0.52 | 0.53 |
| Na_U | 2018 | 419 | west | 0.23 | 0.35 | 0.30 | 0.39 | 0.56 | 0.51 |
| Na_U | 2018 | 419 | west | 0.27 | 0.35 | 0.24 | 0.38 | 0.56 | 0.51 |
| Na_U | 2017 | 419 | east |  | 0.34 | 0.25 | 0.27 | 0.41 | 0.43 |
| Na_U | 2017 | 419 | east |  | 0.37 | 0.37 | 0.29 | 0.36 | 0.42 |
| Na_U | 2017 | 419 | east |  | 0.32 | 0.32 | 0.28 | 0.47 | 0.37 |
| Na_U | 2018 | 419 | east | 0.31 | 0.44 | 0.36 | 0.54 | 0.74 | 0.62 |
| Na_U | 2018 | 419 | east | 0.28 | 0.51 | 0.34 | 0.48 | 0.66 | 0.66 |
| Na_U | 2018 | 419 | east | 0.35 | 0.47 | 0.36 | 0.55 | 0.71 | 0.63 |
| Na_O | 2017 | 650 | west |  | 0.35 | 0.37 | 0.42 | 0.31 | 0.35 |
| Na_O | 2017 | 650 | west |  | 0.36 | 0.39 | 0.35 | 0.23 | 0.36 |
| Na_O | 2017 | 650 | west |  | 0.31 | 0.37 | 0.35 | 0.38 | 0.41 |
| Na_O | 2018 | 650 | west | 0.17 | 0.15 | 0.23 | 0.18 | 0.22 | 0.37 |
| Na_O | 2018 | 650 | west | 0.22 | 0.14 | 0.22 | 0.23 | 0.31 | 0.38 |
| Na_O | 2018 | 650 | west | 0.22 | 0.16 | 0.21 | 0.17 | 0.29 | 0.25 |
| Na_O | 2017 | 650 | east |  | 0.58 | 0.70 | 0.61 | 0.44 | 0.60 |
| Na_O | 2017 | 650 | east |  | 0.58 | 0.66 | 0.58 | 0.58 | 0.63 |
| Na_O | 2017 | 650 | east |  | 0.50 | 0.63 | 0.54 | 0.69 | 0.60 |
| Na_O | 2018 | 650 | east | 0.37 | 0.35 | 0.52 | 0.34 | 0.45 | 0.56 |
| Na_O | 2018 | 650 | east | 0.33 | 0.31 | 0.45 | 0.40 | 0.42 | 0.57 |
| Na_O | 2018 | 650 | east | 0.30 | 0.38 | 0.45 | 0.42 | 0.36 | 0.55 |

S2_Fig.

|  | FLAV average | | | | |
| --- | --- | --- | --- | --- | --- |
| treatment | 185 | 200 | 214 | 228 | 242 |
| Te-N-ND | 0.041477 | 0.090853 | 0.02048 | 0.149568 | 0.342303 |
| Te-S-ND | 0.286818 | 0.306958 | 0.279523 | 0.388586 | 0.509918 |
| Te-N-ID | 0.127912 | 0.179732 | 0.123125 | 0.26596 | 0.408764 |
| Te-S-ID | 0.317521 | 0.447037 | 0.3418 | 0.448779 | 0.614981 |
| Ep-E-ND | 0.139533 | 0.284686 | 0.334396 | 0.419053 | 0.443452 |
| Ep-W-ND | 0.062108 | 0.197423 | 0.108637 | 0.145213 | 0.249669 |
| Ep-E-ID | 0.388715 | 0.485045 | 0.543331 | 0.528026 | 0.548098 |
| Ep-W-ID | 0.203099 | 0.299371 | 0.348281 | 0.319372 | 0.385467 |
|  | FLAV er.st | | | | |
| treatment | 185 | 200 | 214 | 228 | 242 |
| Te-N-ND | 0.013353 | 0.058435 | 0.019432 | 0.004873 | 0.020286 |
| Te-S-ND | 0.034219 | 0.020869 | 0.025198 | 0.009347 | 0.019856 |
| Te-N-ID | 0.01435 | 0.002192 | 0.012604 | 0.010321 | 0.017214 |
| Te-S-ID | 0.038686 | 0.019419 | 0.028839 | 0.012414 | 0.02069 |
| Ep-E-ND | 0.021179 | 0.028612 | 0.038364 | 0.052751 | 0.009009 |
| Ep-W-ND | 0.010395 | 0.026124 | 0.028283 | 0.019835 | 0.033672 |
| Ep-E-ID | 0.026738 | 0.004269 | 0.045694 | 0.036216 | 0.009814 |
| Ep-W-ID | 0.024499 | 0.019283 | 0.007944 | 0.048227 | 0.031504 |
